# Supplementary material for: MicroRNA-148b Targets the TGF-β Pathway to Regulate Angiogenesis and Endothelial-to-Mesenchymal Transition during Skin Wound Healing
Source: Mol Ther. 2018 May 8;26(8):1996–2007. doi: 10.1016/j.ymthe.2018.05.002 (PMC6094488; doi:10.1016/j.ymthe.2018.05.002)
Supplement: Document S1. Figures S1–S8 [file mmc1.pdf]

YMTHE, Volume *26*

Supplemental Information

MicroRNA-148b Targets the TGF-β Pathway to Regulate Angiogenesis and Endothelial-to-Mesenchymal Transition during Skin Wound Healing

Vladislav Miscianinov, Andrea Martello, Lorraine Rose, Elisa Parish, Ben Cathcart, Tijana Mitić, Gillian A. Gray, Marco Meloni, Ayman Al Haj Zen, and Andrea Caporali

A

#	Gene Name	Context Score	Conservation Score
1.	ROCK1	-0.226	0.963
2.	SMAD2	0.030	0.762
3.	INHBB	-0.375	0.903
4.	SMURF2	-0.267	0.318
5.	ACVR1	-0.237	0.634
6.	ACVR2B	0.008	0.778
7.	TGFB2	-0.279	0.885
8.	LTBP1	-0.210	0.557
9.	NOG	-0.292	0.501

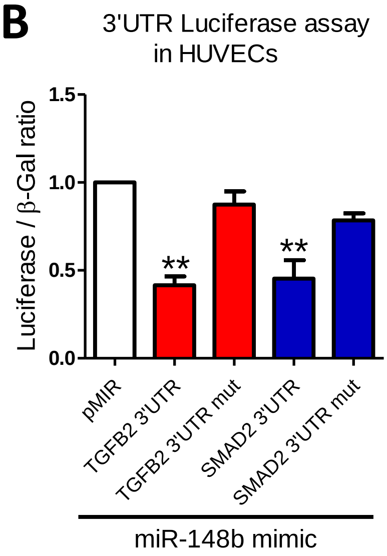

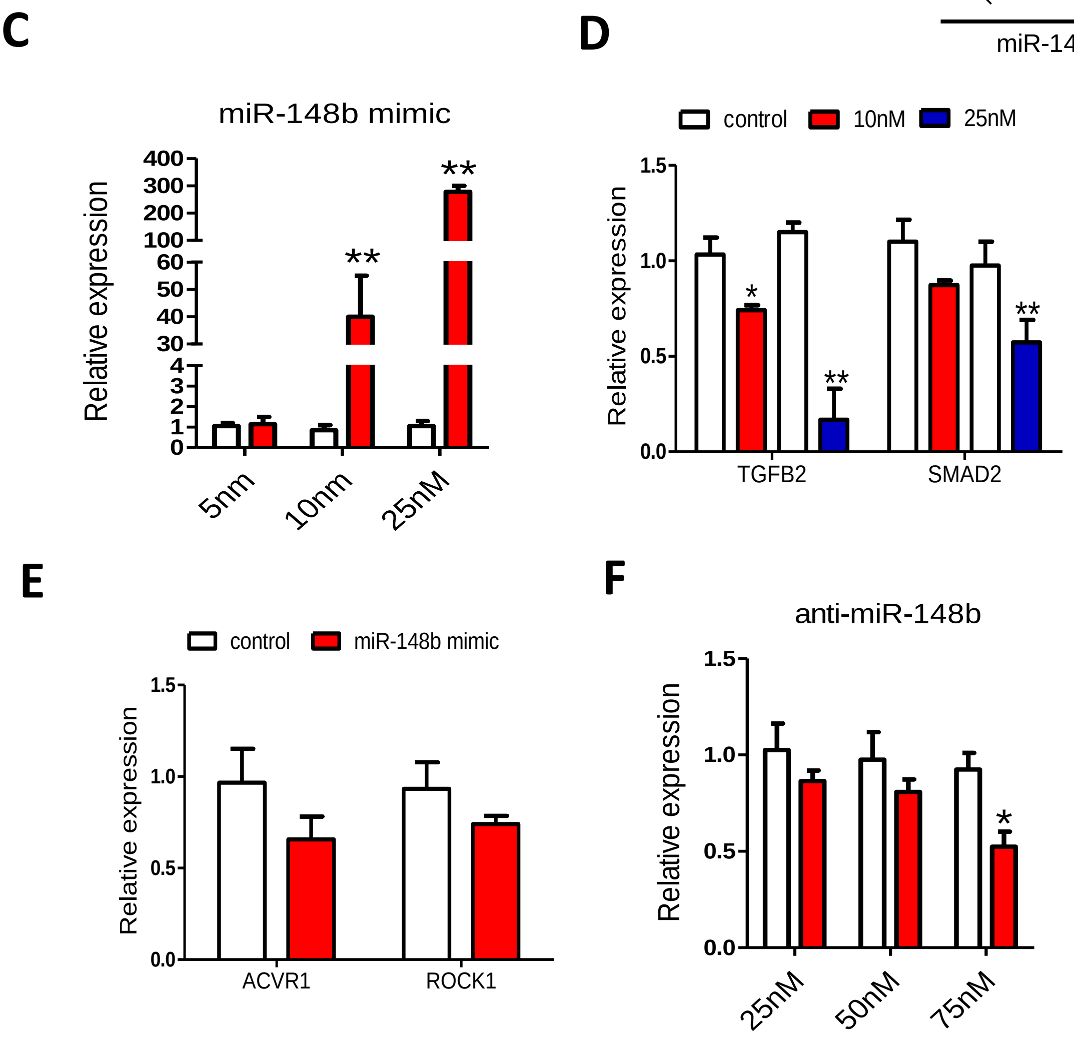

Figure S1. miR-148b *in silico* targets analysis and miR-148b expression level

A. Analysis of miR-148b target genes using miRpath software. Table shows Context Score and Conservation score from Targetscan. **B.** Luciferase activity at 48h post-co-transfection of HUVECs cells with both miR-148b and the following plasmids: 3'-UTR-TGFB2. 3'-UTR-SMAD2 and pMIR as empty plasmid (n=5); **C.** MiR-148b expression levels after miR mimic transfection (n=5); **D.** TGFB2 and SMAD2 expression after different doses of miR-148b mimic transfection (n=5); **E.** ACVR1 and ROCK1 expression miR-148b mimic transfection (n=5); **F.** MiR-148b expression levels after anti-miR-148b transfection (n=5);. Values are means±SEM. *P<0.05; **P<0.01 vs control. Unpaired two-tailed Student's t-test was applied.

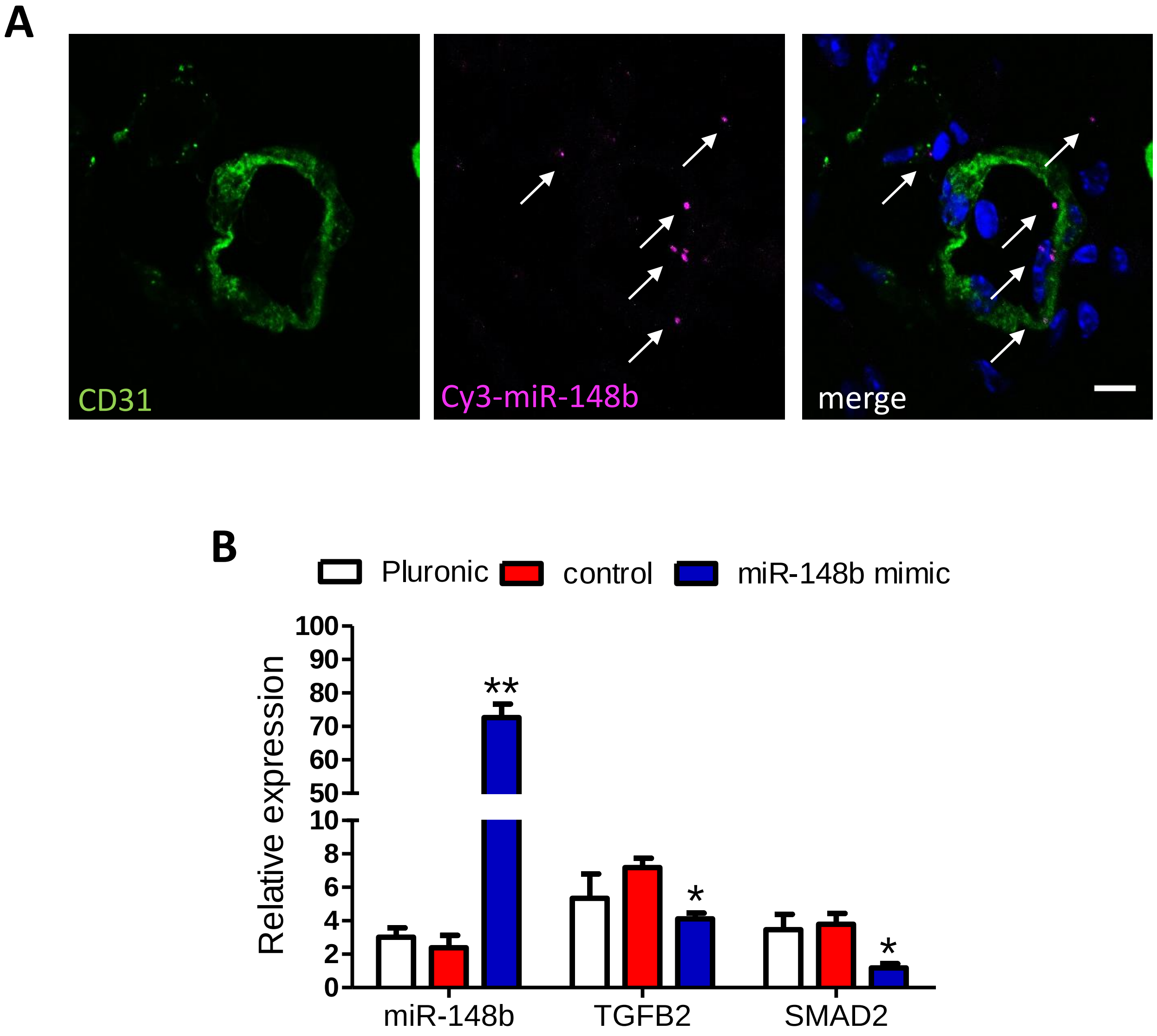

Figure S2: Delivery of miR-148b mimic *in vivo*

A. Representative images of the localization of Cy3 labelled miR-148b (pink dots) in skin wounds. Vessels are stained for CD31 (green), scale bar=25µm (magnification 1000x); **B.** Relative gene expression of miR-148b, *TGFB2* and *SMAD2* in dermal wound after delivering of miR-148b mimic or control oligonucleotides at 7 days (n=5)**;** Values are means±SEM. *P<0.05; **P<0.01 vs control or not-wounded skin. Unpaired two-tailed Student's t-test was applied.

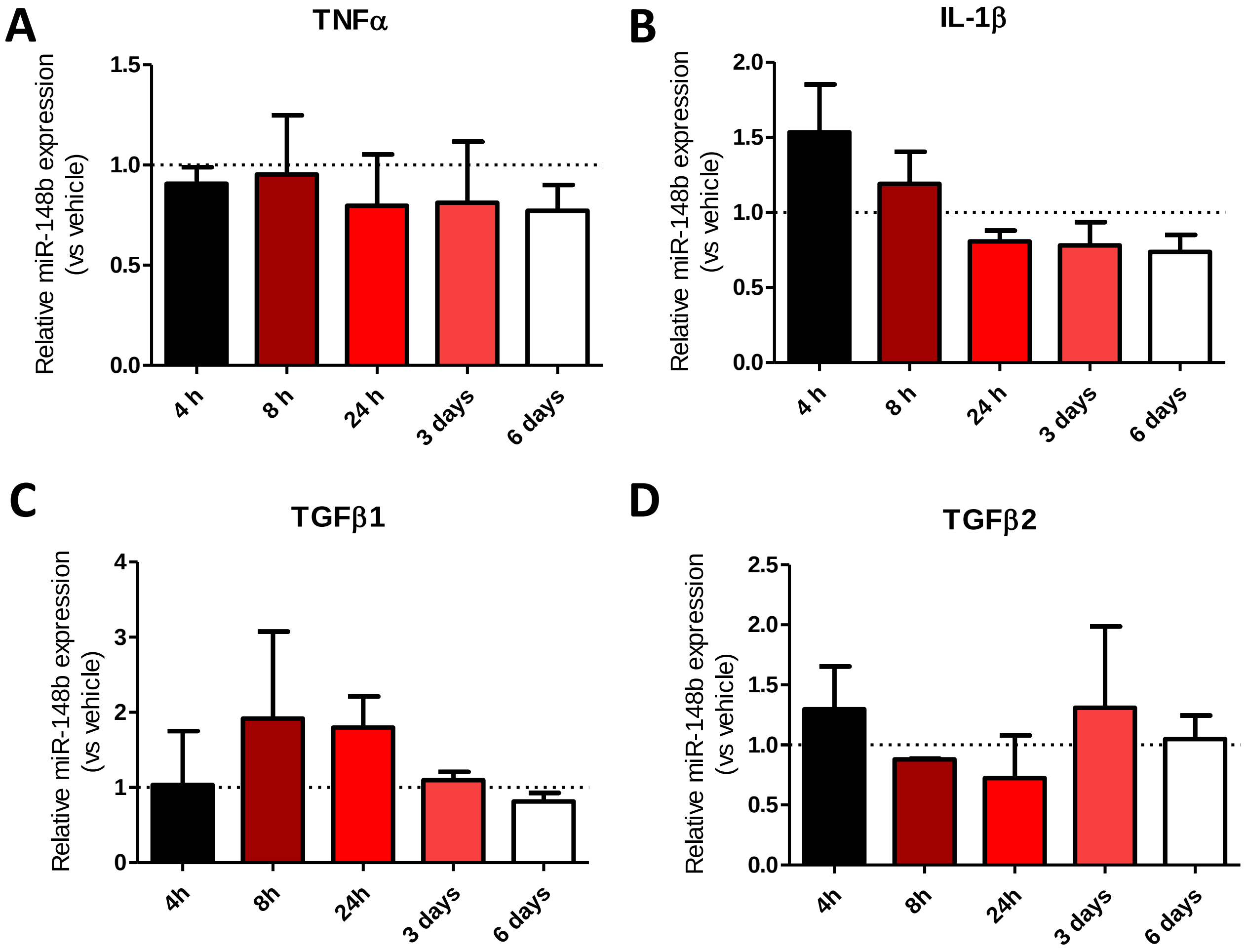

Figure S3: Regulation of miR-148b expression *in vitro*. Relative miR-148b expression after treatment of HUVECs with **A.** TNF-α; **B.** IL-1β; **C.** TGFβ1; **D.** TGFβ2; the dotted line represents relative miR-148b expression in the untreated control HUVEC (n=3); Values are means±SEM.

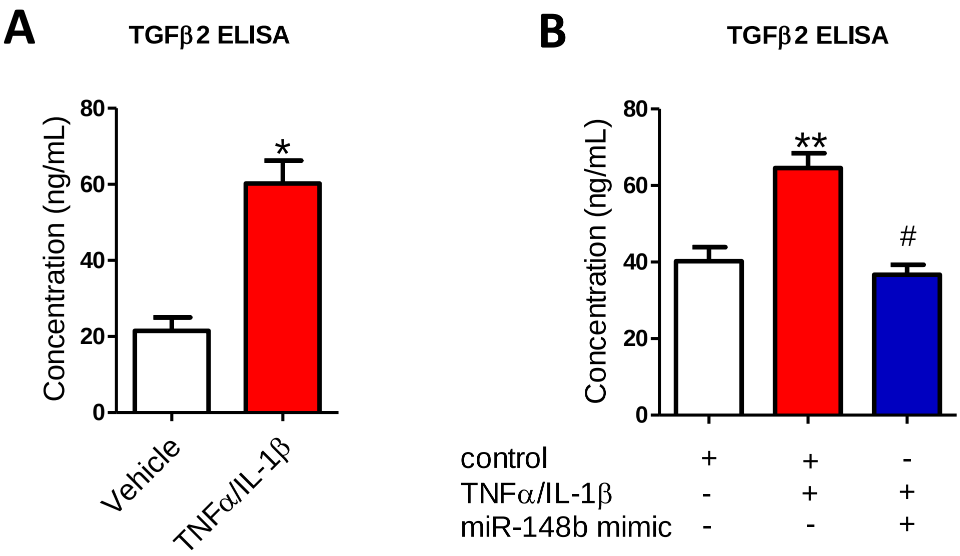

Figure S4. Secreted TGFβ2 following TNFα/IL-1β treatment and miR-148b mimics transfection. **A.** Quantification of TGFβ2 ELISA experiment expressed in TGFβ2 concentration (ng/mL) in TNF-α/IL-1β treated samples vs the control; **B.** Quantification of TGFβ2 ELISA experiment expressed in TGFβ2 concentration (ng/mL) in miR-148b gain-of-function and/or TNFα/IL-1β treated samples vs the control; Values are means±SEM (n=5). *P<0.05; **P<0.01 vs control; . #P<0.05 vs TNFα/IL-1β. Unpaired two-tailed Student's t-test and one-way ANOVA statistical test followed by Bonferroni post-hoc analyses were applied.

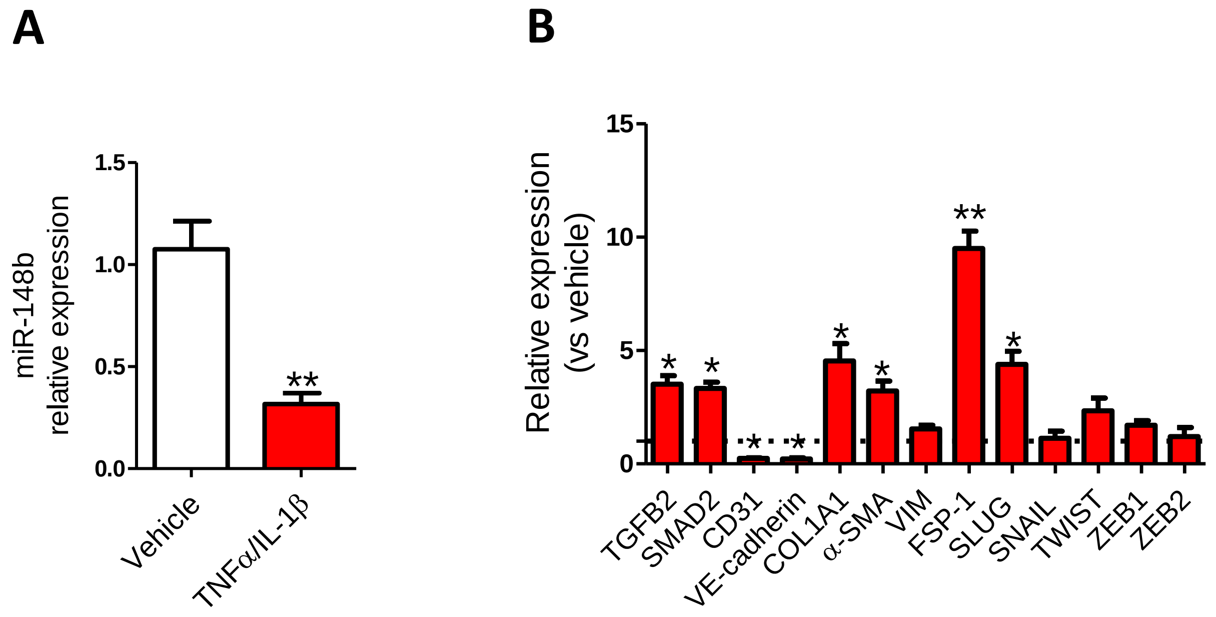

Figure S5: miR-148b EndMT markers expression in ECs after 14 days of cytokine-treatment.
A. Relative miR-148b expression after treatment of HUVECs treated with TNFα/IL-1β for 14 days; **B.** Relative expression of TGFB2, SMAD2, CD31, VE-cadherin, SNAIL, SLUG, TWIST, ZEB1/2, VIM, FSP-1, α-SMA and COL1A1; the dotted line represents relative expression in the vehicle-treated HUVECs (n=3); Values are means±SEM. *P<0.05; **P<0.01 vs vehicle. Unpaired two-tailed Student's t-test was applied.

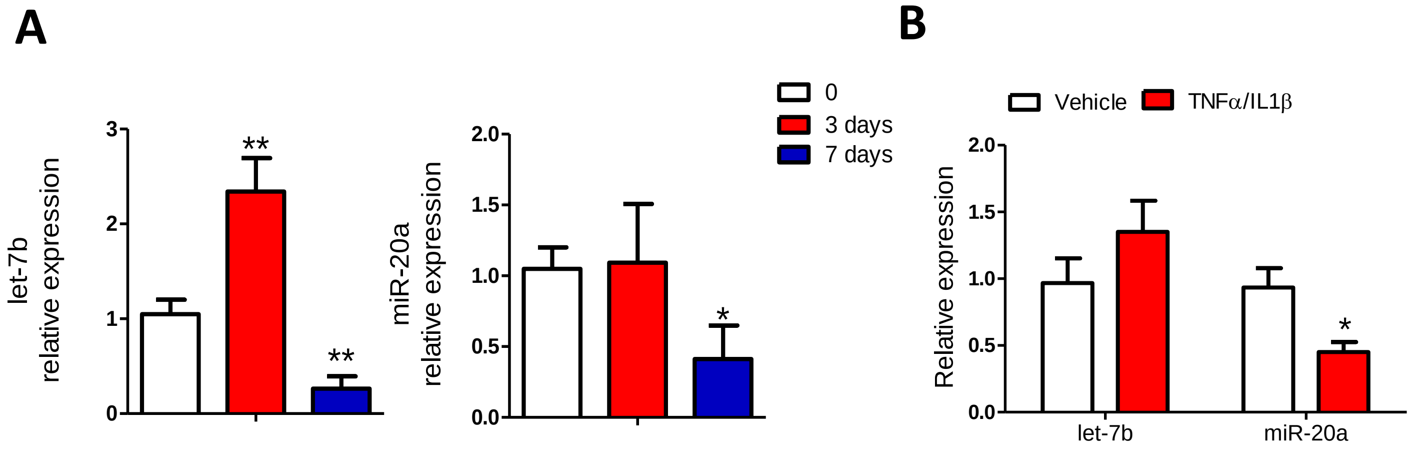

Figure S6: Regulation of let7b and miR-20a expression *in vitro and in vivo*. **A.** Relative let7-b and miR-20a expression in vivo during wound healing progression and **B.** following TNF-α/IL-1β treatment in ECs Values are means±SEM (n=3). *P<0.05; **P<0.01 vs day 0; . *P<0.05 vs vehicle. Unpaired two-tailed Student's t-test was applied.

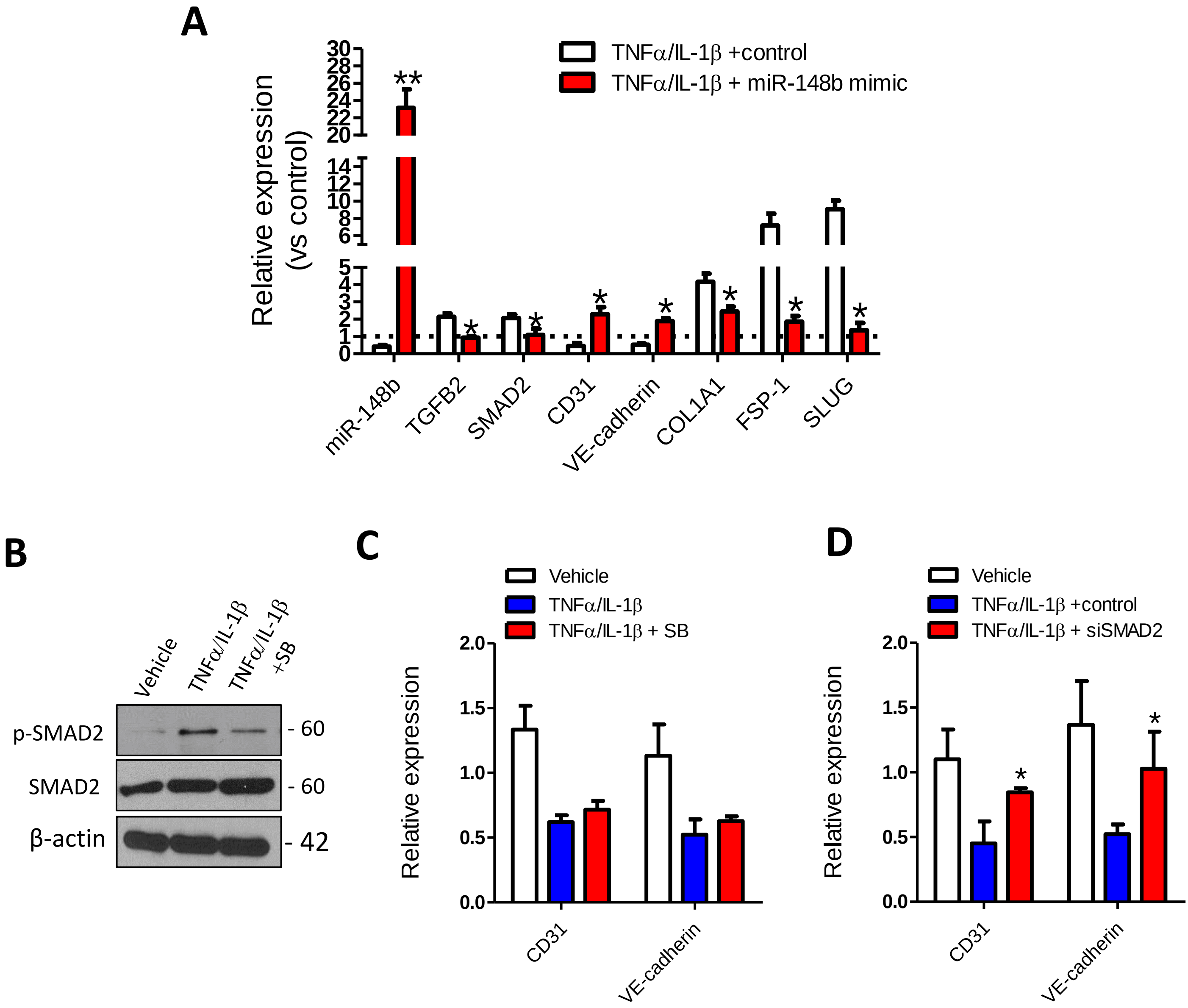

Figure S7: miR-148b mimics and SMAD2 siRNA regulate cytokine-mediated EndMT
HUVECs were transfected with miR-148b mimic, SMAD2 siRNA or control and treated with TNF-α/IL-1β for 6 days. **A.** Relative expression of miR-148b, TGFB2, SMAD2, CD31, VE-cadherin, COL1A1, FSP-1 and SLUG (n=3); **B.** Western blot for phospho-SMAD2, SMAD2 and β-actin in HUVECs treated with TNF-α/IL-1β and ALK5 inhibitor (SB431542; 10μM) for 6 days. **C.** Relative expression CD31 and VE-Cadherin in HUVECs following treatment with ALK5 inhibitor or **D.** SMAD2 siRNA and/or TNFα/IL-1β ; Values are means±SEM (n=3). *P<0.05; **P<0.01 vs TNFα/IL1β + control oligonucleotides. Unpaired two-tailed Student's t-test was applied.

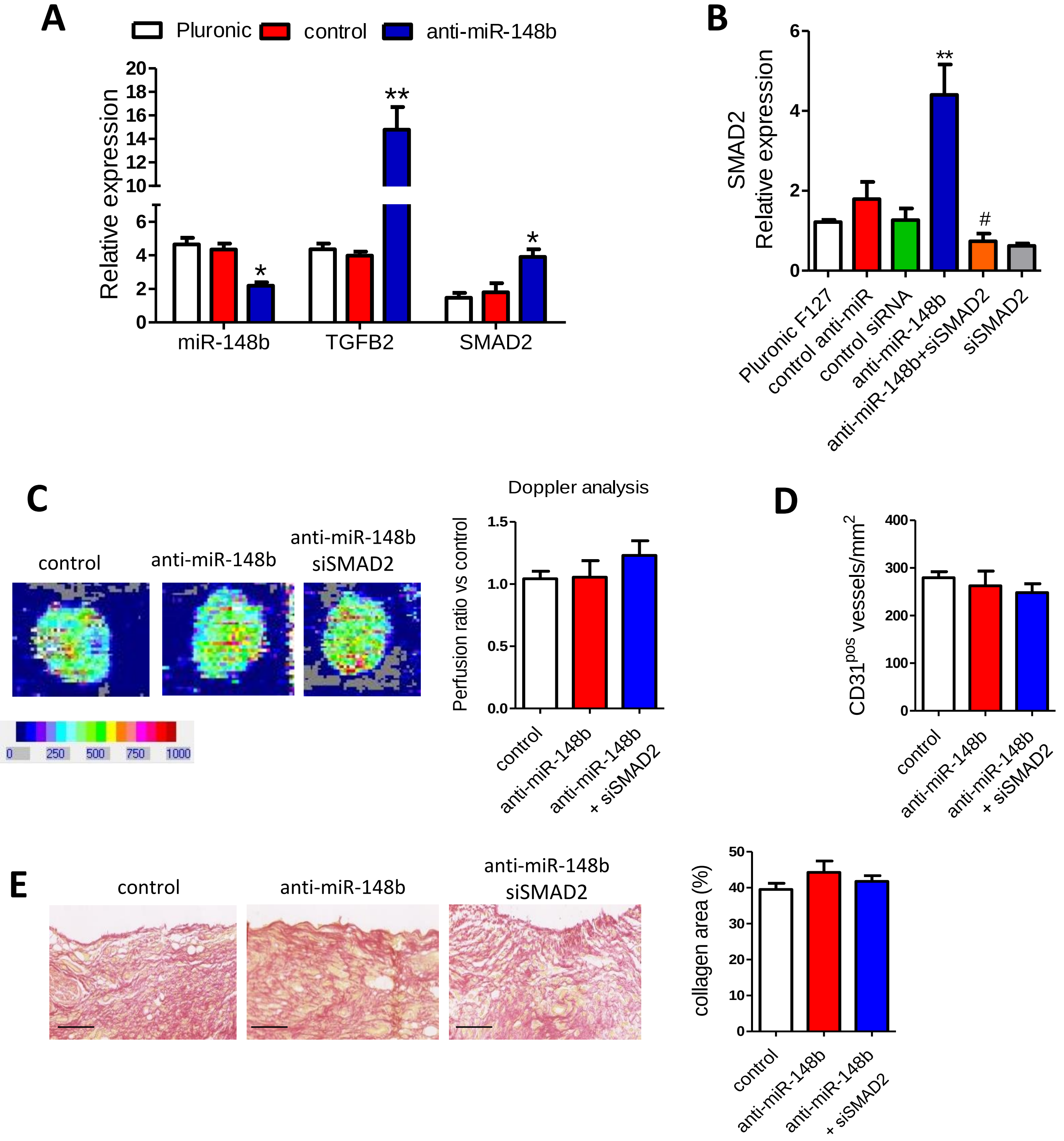

Figure S8: Analysis of anti-miR-148b delivery *in vivo*

Dermal wounds were treated with control and anti-miR-148b or SMAD2 siRNA at 7 days. **A.** Relative expression of miR-148b, TGFB2 and SMAD2 (n=5); **B.** Relative SMAD2 expression in dermal wounds treated (n=5); **C.** *left* Representative colour laser Doppler images are taken at 5 days post wounds. *Right.* Chart shows level of wounds perfusion in mice (calculated as the ratio between treated and control blood flow; n=8 per group). **D**. Quantification of vessel density expressed as CD31 positive vessels/mm2 (n=8); **E.** *left*, Sirius Red staining of wounds obtained at day 7 post wounding; *right*, Collagen quantification. Scale bar represents 100µm (magnification 200x). Values are means±SEM. *P<0.05; **P<0.01 vs control; $^{\#}$P<0.05 vs anti-miR-148b . Unpaired two-tailed Student's t-test and one-way ANOVA statistical test followed by Bonferroni post-hoc analyses were applied.
