# Supplementary material for: MicroRNA-148b Targets the TGF-β Pathway to Regulate Angiogenesis and Endothelial-to-Mesenchymal Transition during Skin Wound Healing
Source: Mol Ther. 2018 May 8;26(8):1996–2007. doi: 10.1016/j.ymthe.2018.05.002 (PMC6094488; doi:10.1016/j.ymthe.2018.05.002)
Supplement: Document S2. Article plus Supplemental Information [file mmc2.pdf]

Molecular Therapy
Original Article

MicroRNA-148b Targets the TGF-β Pathway to Regulate Angiogenesis and Endothelial-to-Mesenchymal Transition during Skin Wound Healing

Vladislav Miscianinov,[1] Andrea Martello,[1] Lorraine Rose,[1] Elisa Parish,[1] Ben Cathcart,[1] Tijana Mitić,[1] Gillian A. Gray,[1] Marco Meloni,[1] Ayman Al Haj Zen,[2] and Andrea Caporali[1]

[1]University/British Heart Foundation Centre for Cardiovascular Science, QMRI, University of Edinburgh, Edinburgh EH16 4TJ, UK; [2]British Heart Foundation Centre of Research Excellence, Wellcome Trust Centre for Human Genetics, Division of Cardiovascular Medicine, Radcliffe Department of Medicine, University of Oxford, Oxford OX3 7BN, UK

Transforming growth factor beta (TGF-β) is crucial for regulation of the endothelial cell (EC) homeostasis. Perturbation of TGF-β signaling leads to pathological conditions in the vasculature, causing cardiovascular disease and fibrotic disorders. The TGF-β pathway is critical in endothelial-to-mesenchymal transition (EndMT), but a gap remains in our understanding of the regulation of TGF-β and related signaling in the endothelium. This study applied a gain- and loss-of function approach and an *in vivo* model of skin wound healing to demonstrate that miR-148b regulates TGF-β signaling and has a key role in EndMT, targeting *TGFB2* and *SMAD2*. Overexpression of miR-148b increased EC migration, proliferation, and angiogenesis, whereas its inhibition promoted EndMT. Cytokine challenge decreased miR-148b levels in ECs while promoting EndMT through the regulation of SMAD2. Finally, in a mouse model of skin wound healing, delivery of miR-148b mimics promoted wound vascularization and accelerated closure. In contrast, inhibition of miR-148b enhanced EndMT in wounds, resulting in impaired wound closure that was reversed by SMAD2 silencing. Together, these results demonstrate for the first time that miR-148b is a key factor controlling EndMT and vascularization. This opens new avenues for therapeutic application of miR-148b in vascular and tissue repair.

INTRODUCTION

It is well known that the transforming growth factor beta (TGF-β) signaling pathway plays a crucial role in the regulation of vascular function in health and disease.[1] During canonical activation of the TGF-β pathway, TGF-β binds to the heteromeric receptor complex formed by the activin-like kinase 5 (ALK5, also known as the TGF-β type I receptor, TGFBR1) and the TGF-β type II receptor (TGFBR2). Phosphorylation of ALK5 by TGFBR2 kinase activates its catalytic kinase domain allowing the activation of the receptor-regulated Smads (i.e., SMAD2 and SMAD3), which translocate to the nucleus, where they regulate the transcription of specific target genes.[2]

Activation of the TGF-β pathway in endothelial cells (ECs) during angiogenesis is reported to have pro-angiogenic, as well as anti-angiogenic effects. Discrepancies might be explained by the variations in ligand concentration, culture conditions, cell type, and developmental or disease stage *in vivo*. In addition, the effects of TGF-β signaling depend on the interplay between different members of the TGF-β family.[3] Accumulating evidence over the past few years is revealing the role of the TGF-β pathway in regulation of cellular plasticity, thus including the control of EC phenotype.[4] EC plasticity is represented by the endothelial-to-mesenchymal transition (EndMT), allowing the endothelium to not only alter its fate but to provide a potentially important additional source of mesenchymal cells for participation in fibrosis.[5,6] During the EndMT process, ECs lose their endothelial specification and acquire a mesenchymal-like phenotype. Specifically, loss of vascular endothelial cadherin (VE-cadherin) and platelet-EC adhesion molecule 1 (PECAM-1, CD31) are observed along with elevated expression of α-smooth muscle actin (α-SMA), vimentin (VIM), N-cadherin, and extracellular matrix (ECM) proteins like collagen type I and III.[7] ECs undergoing EndMT also exhibit cytoskeletal rearrangement, resulting in a change in cell polarity whereby they acquire a stretched and more fibroblast-like morphology.[7] Recently, EndMT has been reported to play an important role during the embryonic stages of cardiac and pulmonary artery development.[8,9] In addition, EndMT has a pathological role through promoting tumor growth and progression,[10] contributing to cardiac[11] and renal fibrosis[12] as well as vascular remodelling[13] and atherosclerosis.[14]

MicroRNAs (miRNAs) are post-transcriptional inhibitory regulators of gene expression that bind to complementary mRNA transcripts.[15] Their capacity to simultaneously inhibit many different mRNAs

Received 22 September 2017; accepted 4 May 2018;
https://doi.org/10.1016/j.ymthe.2018.05.002.

Correspondence: Andrea Caporali, PhD, University/British Heart Foundation Centre for Cardiovascular Science, The Queen's Medical Research Institute, University of Edinburgh, Edinburgh EH16 4TJ, UK.
E-mail: a.caporali@ed.ac.uk

 © 2018 The Author(s).
This is an open access article under the CC BY license (http://creativecommons.org/licenses/by/4.0/).

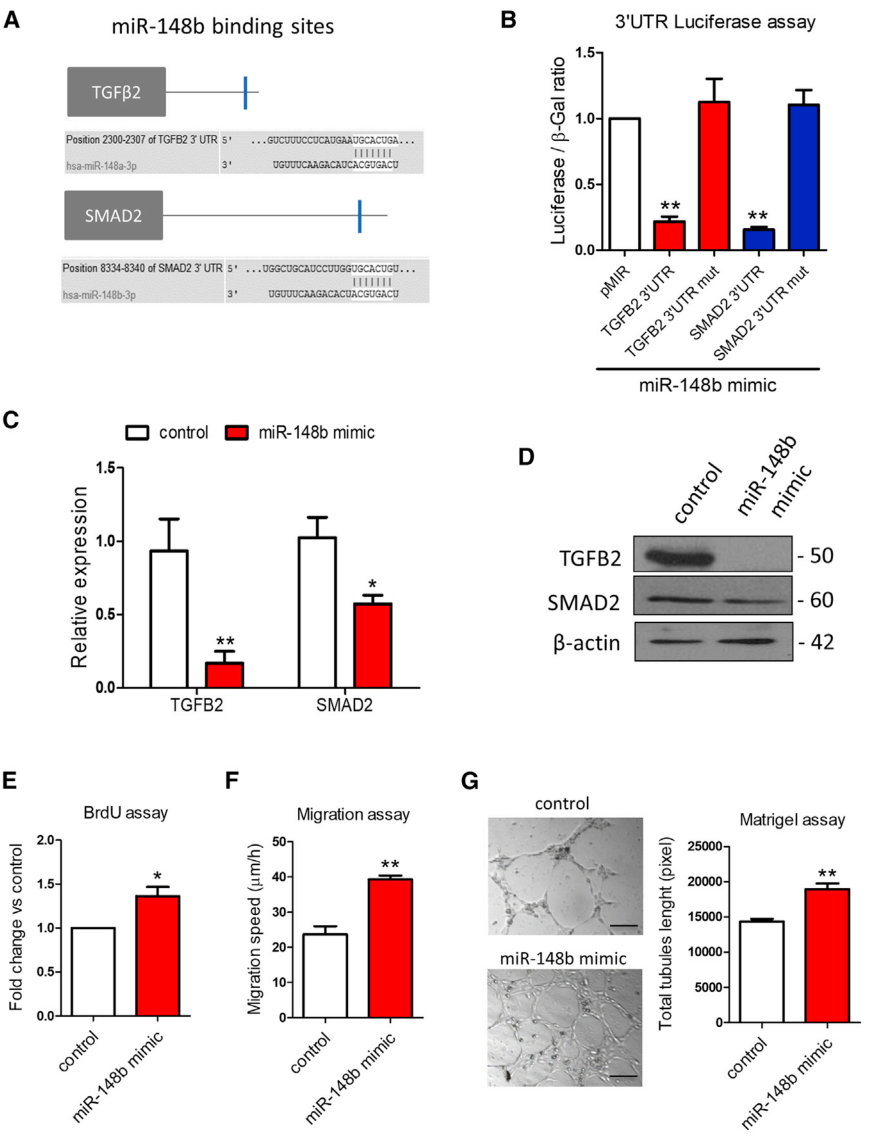

Figure 1. TGFB2 and SMAD2 Are miR-148b Target Genes

(A) *In silico* analysis of TGFB2 and SMAD2 3′ UTR with TargetScan identifies putative miR-148b-binding sites (blue). (B) Luciferase activity at 48 hr post-co-transfection of HEK293T cells with both miR-148b and the following plasmids: 3′ UTR-TGFB2, 3′ UTR-SMAD2, and pMIR as empty plasmid (n = 5). (C) Relative gene expression of TGFB2 and SMAD2 after miR-148b overexpression (n = 3). (D) Western blot analysis of TGFB2 and SMAD2 in samples transfected with miR-148b mimic versus control; β-actin is used as a loading control (n = 3); HUVECs were transfected with miR-148b mimic or control oligonucleotides for 48 hr. (E) EC proliferation was analyzed by BrdU incorporation assay (n = 5). (F) EC migration reported as migration speed (μm/hr) (n = 5). (G) Representative Matrigel assay images and quantification as total tubule length (n = 5); values are means ± SEM. *p < 0.05; **p < 0.01 versus control and pMIR vector in experiment b. Unpaired two-tailed Student's t test was applied.

allows for efficient amplification of biological responses.[16] Recent studies have revealed important roles for miRNAs as therapeutic tools in regulating physiological and pathological angiogenesis, through the regulation of EC function (reviewed in Caporali and Emanueli[17,18]).

Several miRNAs have been identified that target elements of the TGF-β/Smad signaling axis[19] and, more recently, contribute to the EndMT process.[20] Although the role of TGF-β signaling in the regulation and maintenance of adult EC homeostasis and plasticity is now well established,[2] less is known about the mechanisms that regulate the TGF-β signaling pathway. Using miRNA target-prediction analysis, we have found that miR-148b targets are strongly enriched for the components of TGF-β pathway. The present study aims to investigate how this miRNA regulates EC function and how it impacts on TGF-β signaling in these cells. miR-148b, together with miR-148a and miR-152, comprises the miR-148/152 family.[21] Although aberrant expression of the miR-148/152 family has been observed in tumors, and in non-tumor diseases such as immunoglobulin A (IgA) nephropathy[22] and atherosclerotic lesions;[23] its function has not been explored in ECs.

Here, we establish that in ECs, miR-148b plays a critical role in promoting angiogenesis and that its inhibition promotes EndMT process through regulation of the target genes, *TGFB2* and *SMAD2*. Furthermore, we demonstrate, in an *in vivo* model of wound healing, that overexpression of miR-148b increases vascularization and accelerates wound closure, whereas miR-148b inhibition leads to an enhanced EndMT in the wound, resulting in impaired closure.

RESULTS

miR-148b Targets TGFB2 and SMAD2 and Regulates EC Functions

Gene set enriched analysis (miRpath[24]) has identified components of the TGF-β pathway as putative targets of miR-148b (Figure S1A). Moreover, the TargetScan algorithm[25] predicted *TGFB2* and *SMAD2* to be direct targets of miR-148b (Figure 1A). Accordingly, both gene targets were predicted to contain a single conserved binding sequence for miR-148b in their 3′ UTR (Figure 1A). To investigate whether miR-148b directly binds the 3′ UTR of *TGFB2* and *SMAD2*, we performed a luciferase reporter assay in which luciferase reporter gene was fused to the wild-type 3′ UTR of *TGFB2* or *SMAD2*,

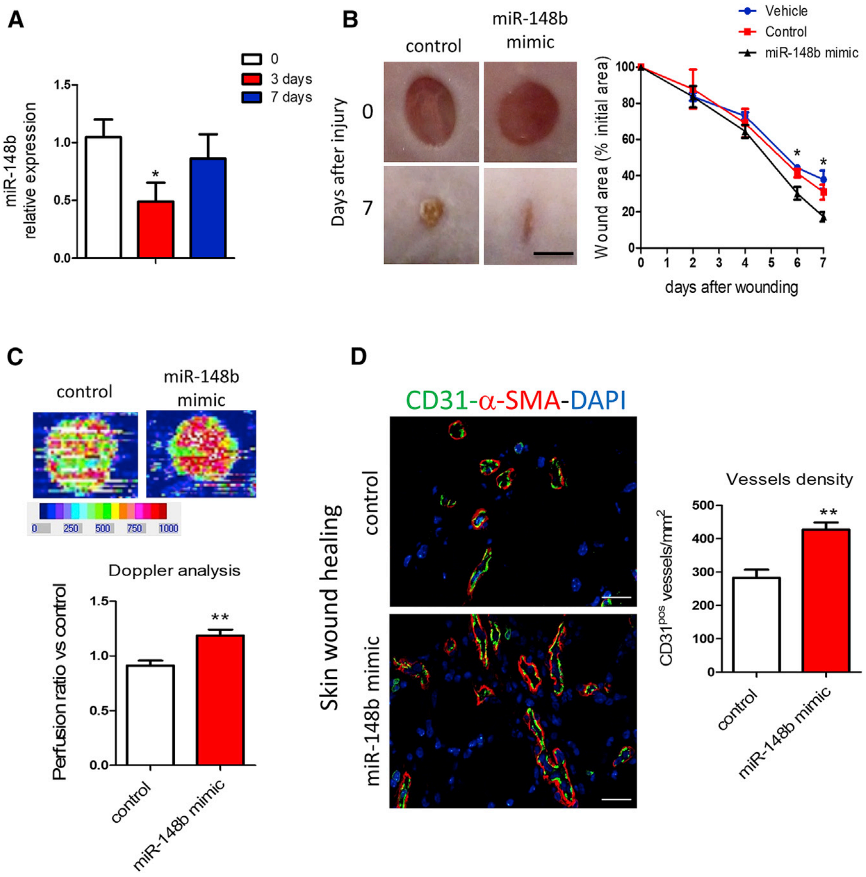

Figure 2. miR-148b Regulates Angiogenesis and Accelerates Wound Healing

(A) Expression of miR-148b at 3 and 7 days after wounding, relative to unwounded skin (n = 5). (B) Left, representative images of control and miR-148b mimic-treated wounds at 0 and 7 days post-wound-injury; scale bar, 5 mm. Right, the level of wound closure is expressed as a percentage of initial wound area (n = 8). (C) Representative color laser Doppler images are taken at 5 days post-wounding. The chart shows the level of wound perfusion in mice (calculated as the ratio between treated and control blood flow; n = 8 per group). (D) Left, immunohistochemistry for CD31 and α-SMA in miR-148b mimic-treated or control-oligonucleotide-treated skin wounds; scale bars, 100 μm (magnification 400×). Right, quantification of vessel density expressed as CD31-positive vessels/mm^2 (n = 8). Values are means ± SEM. *$p < 0.05$; **$p < 0.01$ versus control. Unpaired two-tailed Student's t test was applied.

respectively. Overexpression of miR-148b decreased luciferase activity for each of the putative target genes, whereas mutation of the binding sites restored it (Figures 1B and S1B). In addition, miR-148b mimics, at the dose of 25 nM, reduced both target gene mRNA (Figures 1C, S1C, and S1D) and protein levels (Figure 1D). Notably, miR-148b upregulation did not affect the expression of *ACVR1* and *ROCK1*, previously identified target genes of miR-148/152 cluster[26,27] (Figure S1E). We then investigated the role of miR-148b on the functional properties of ECs. miR-148b overexpression significantly increased the proliferation of human umbilical vein EC (HUVECs), as demonstrated by bromodeoxyuridine (BrdU) incorporation (Figure 1E). The migration capacity of HUVECs transfected with a miR-148b mimic was also significantly elevated (Figure 1F), along with their ability to form tubes in an *in vitro* Matrigel assay (Figure 1G).

miR-148b Promotes Angiogenesis and Accelerates Skin Wound Healing

To follow up the *in vitro* results, we asked whether miR-148b could influence angiogenesis in a mouse model of wound healing. In this model, following skin puncture, the peak of neovascularization occurs between days 4 and 8 after wounding. Vessel density generally returns to initial levels around 3 weeks later. Thus, before, and at the peak of vascularization, this model characterizes the angiogenic response.[28]

Three days after wound injury, the expression of miR-148b was found to be downregulated, going back to basal level after 7 days (Figure 2A). The analysis of Cy3-labeled miR-148b mimics in the wounds demonstrated that the mimics could reach the endothelium *in vivo* (Figure S2A).

Topical delivery of miR-148b mimics starting at day 3 after wounding increased the expression of miR-148b and decreased the expression levels of both targets at day 7 after wounding (Figure S2B). Moreover, miR-148b overexpression also accelerated wound closure (Figure 2B) and led to a significant increase in wound perfusion detected by Doppler analysis (Figure 2C) and vessel density (Figure 2D). These data indicate that treatment with miR-148b mimics promotes angiogenesis and accelerates wound healing in *vivo*.

Loss of Function of miR-148b Promotes Endothelial-Mesenchymal Transition

To analyze the phenotypic effect of miR-148b loss-of-function on ECs, HUVECs were transfected with anti-miR-148b oligonucleotides (Figure S1F). The inhibition of miR-148b expression decreased the proliferation of HUVECs (Figure 3A); however, it did not cause a change in EC migration or tubulogenesis (Figures 3B and 3C). Next, we examined the capacitance of the EC membrane, changes in which reflect structural changes in the plasma membrane and rearrangement of the cytoskeleton (the capacitance of ECs is ~1.1 μF/cm^2, whereas for mesenchymal cells, it is ~3.0 μF/cm^2).[29] Knockdown of miR-148b in ECs increased the membrane capacitance, consistent with the occurrence of transition from an endothelial-to-mesenchymal phenotype (Figure 3D). In line with this, miR-148b loss-of-function increased the expression of its target genes, *TGFB2* and *SMAD2* and the expression of collagen 1A1

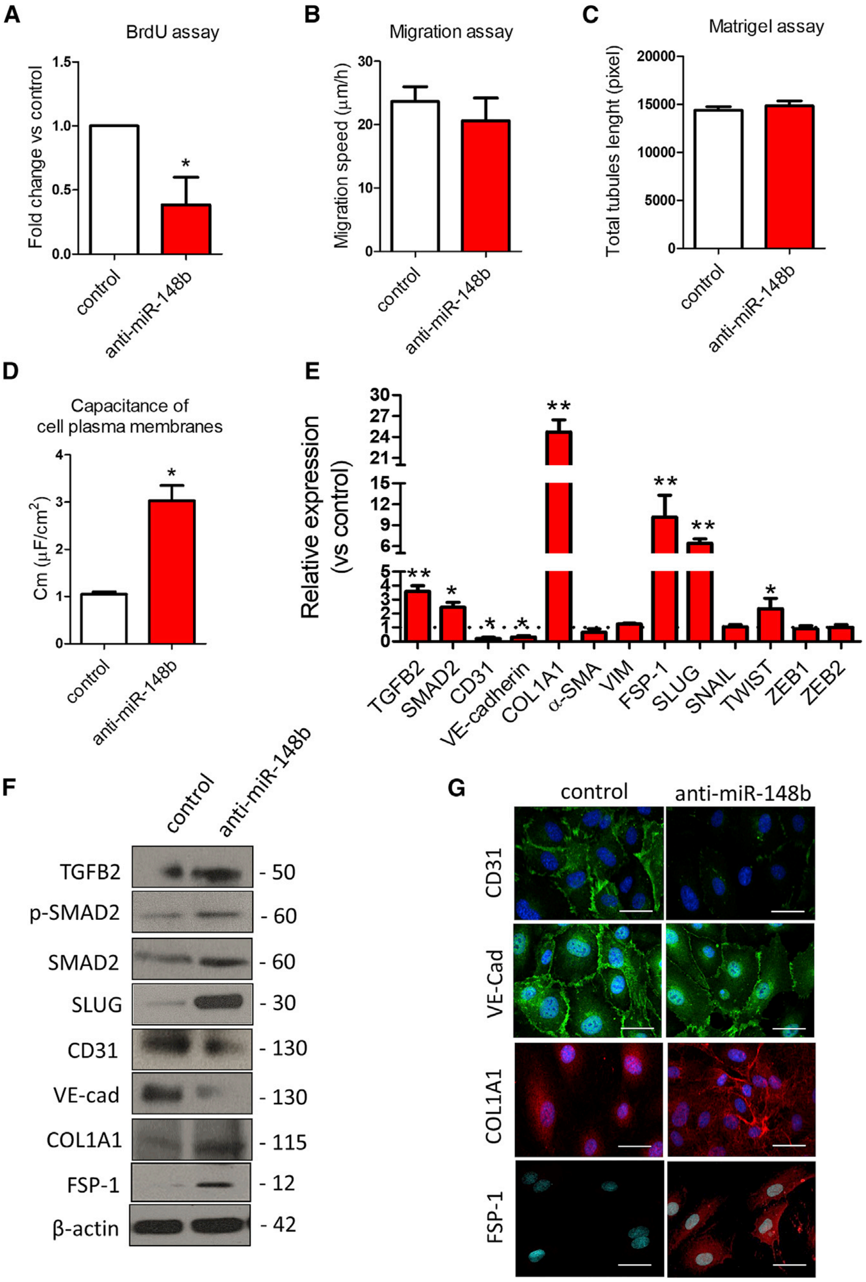

Figure 3. Inhibition of miR-148b Induces EndMT

HUVECs were transfected with anti-miR-148b or control oligonucleotides for 48 hr. (A) EC proliferation was analyzed by BrdU incorporation assay (n = 5). (B) EC migration reported as migration speed (μm/hr) (n = 5). (C) Matrigel assay quantification as total tubules length (n = 5). (D) Quantification of cell membrane capacitance in HUVEC knockdown for miR-148b or control (n = 5). (E) Relative gene expression of TGFB2, SMAD2, CD31, VE-cadherin COL1A1, SNAIL, SLUG, TWIST, ZEB1/2, VIM, FSP-1, and α-SMA (n = 3). (F) Western blot analysis of TGFB2, SMAD2, p-SMAD2, SLUG, CD31, VE-cadherin, COL1A1, and FSP-1; β-actin is used as a loading control (n = 3). (G) Immunofluorescence images showing localization of CD31, VE-cadherin, COL1A1, and FSP-1 in anti-miR-148b- or control-transfected cells (n = 3); scale bars, 50 μm (magnification 400×). Values are means ± SEM. *p < 0.05; **p < 0.01 versus control. Unpaired two-tailed Student's t test was applied.

(*COL1A1*) and fibroblast-specific protein 1 (*FSP-1*) but decreased the expression of *CD31* and *VE-Cadherin*, without detectable changes in *α-SMA* or *VIM* levels (Figure 3E). Moreover, expression analysis of the transcription factors involved in EndMT, such as *SNAIL*, *SLUG*, *TWIST*, and *ZEB1/2*, showed strong upregulation of SLUG after miR-148b inhibition (Figure 3E). Western blot analysis confirmed the upregulation of TGFB2, SMAD2 and its phosphorylation, the appearance of mesenchymal marker COL1A1, FSP-1, along with suppression of CD31 and endothelial VE-cadherin and the upregulation of SLUG in ECs lacking miR-148b (Figure 3F). Furthermore, immunofluorescence staining revealed that control-treated HUVECs display a typically rounded/cobblestone morphology that was lost in anti-miR-148b-treated cells. Anti-miR-148b treatment also resulted in a pronounced decrease in CD31 and VE-cadherin and increase in COL1A1 and FSP-1 immunoreactivity (Figure 3G). Taken together, these data show that loss of miR-148b function leads to EndMT.

Pro-inflammatory Cytokines Promote EndMT via Downregulation of miR-148b

It has been reported that EndMT is closely linked to inflammation and high levels of pro-inflammatory cytokines.[30] To investigate whether cytokine treatment could reduce the expression of miR-148b, thus triggering EndMT, we treated HUVECs with TNF-α, interleukin-1β (IL-1β), TGF-β1, or TGF-β2, as well as with a combination of cytokines for 6 days (Figures S3A–S3D). Interestingly, we found that only the combination of TNF-α- and IL-1β-inhibited miR-148b expression after 3 days, with its strongest downregulation at 6 days (Figure 4A). This was accompanied by significantly increased expression of TGFB2 and SMAD2, at both mRNA and protein levels (Figures 4B and 4C), phosphorylation of SMAD2 (Figure 4C), and increased secretion of TGF-β2 (Figure S4A). To test whether cytokine-induced miR-148b downregulation governs EndMT, we analyzed endothelial- and fibroblast-specific markers after the treatment with TNF-α/IL-1β. Consistent with enhancement of EndMT, both mRNA and protein expression of the endothelial markers CD31 and VE-cadherin were decreased (Figures 4B and 4C). These findings were further

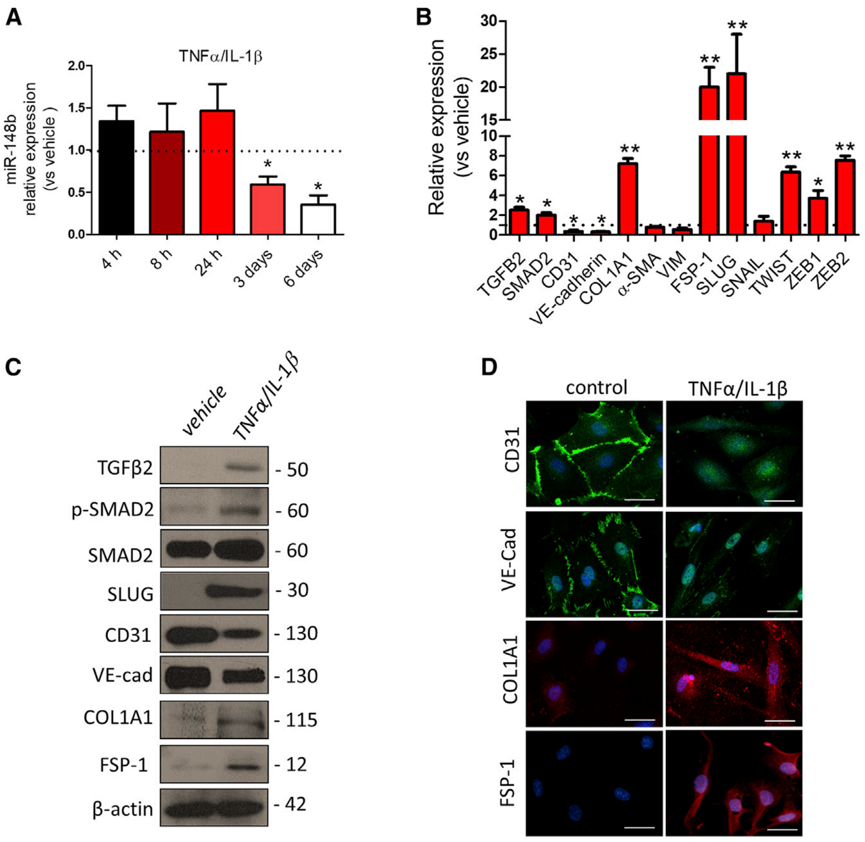

Figure 4. Chronic Inflammation Downregulates miR-148b in ECs

HUVECs were treated with a combination of TNF-α and IL-1β for 6 days. (A) miR-148b expression levels after TNF-α/IL-1β treatment. (B) Relative expression of miR-148b, TGFB2, SMAD2, CD31, VE-cadherin, SNAIL, SLUG, TWIST, ZEB1/2, VIM, FSP-1, α-SMA, and COL1A1. (C) Western blot analysis of TGFB2, p-SMAD2, SMAD2, CD31, VE-cadherin, SLUG, FSP-1, and COL1A1; β-actin was used as a loading control. (D) Immunofluorescence images showing localization of CD31, VE-cadherin, COL1A1, and FSP-1 in TNF-α/IL-1β-treated cells; scale bars, 50 μm (magnification 400×). Values are means ± SEM. *p < 0.05; **p < 0.01 versus vehicle. Unpaired two-tailed Student's t test was applied.

confirmed by immunofluorescence staining of HUVECs treated with TNF-α/IL-1β (Figure 4D). In contrast, mRNA expression and protein levels of fibroblast-specific COL1A1 and FSP-1 (Figures 4B and 4C) were increased 6 days after TNF-α/IL-1β treatment, corroborated by an increase in cellular and extracellular immunofluorescence signals (Figure 4D). Moreover, upregulation of SLUG was confirmed at mRNA and protein levels in cytokine-treated ECs (Figures 4B and 4C). Notably, HUVECs cultured for 14 days in the presence of TNF-α/IL-1β maintained the downregulation of miR-148b and acquired expression of α-SMA, in addition to other markers of EndMT (Figures S5A and S5B).

Finally, we analyzed the expression of let-7b and miR-20a, miRNAs involved in the EndMT process.[31,32] While miR-20a and let-7b were both downregulated *in vivo* during wound healing (Figure S6A), only miR-20a was downregulated by cytokine treatment in ECs (Figure S6B).

miR-148b Overexpression Rescues Cytokine-Mediated EndMT

We next examined whether miR-148b gain of function could rescue the endothelial phenotype and reduce cytokine-induced EndMT. In response to a miR-148b mimic, we observed a decreased in TGFB2, SMAD2, SLUG, FSP-1, COL1A1, and a reduced phosphorylation of SMAD2 in HUVECs treated with TNF-α/IL-1β, whereas expression of CD31 and VE-cadherin at mRNA and protein levels was rescued (Figures S4B, S7A, and 5A). The maintenance of endothelial markers CD31 and VE-cadherin on EC membrane and the decrease of COL1A1 and FSP-1 staining was also confirmed by immunostaining (Figure 5B).

To better understand the contribution of target genes in this model of EndMT, we interfered with the TGF-β pathway using a TGF-β receptor (ALK5) inhibitor (SB431542) or small interfering RNA (siRNA) for SMAD2. Knockdown of SMAD2 in HUVECs treated with cytokines partially restored *CD31* and *VE-cadherin* expression, whereas blocking of ALK5 reduced the level of SMAD2 phosphorylation but did not rescue endothelial marker expression (Figures S7B and S7C). We next confirmed at protein level that the SMAD2 siRNA partially restores the level of CD31 and VE-cadherin in cytokine-treated ECs, whereas decreased SLUG, FSP-1, and COL1A1 (Figure 5C). Changes in expression of CD31 and VE-cadherin, FSP-1, and COL1A1 were further confirmed by immunocytochemistry (Figure 5D).

miR-148b Loss of Function Attenuates Wound Repair by Enabling EndMT in a Skin-Wound-Healing Model

Next, we investigated whether miR-148b-mediated EndMT also occurs *in vivo* in the mouse model of skin wound healing. Delivery of anti-miR-148b or control oligonucleotides immediately after wounding and every 2 days thereafter resulted in increased mRNA expression of *TGFB2* and *SMAD2* targets in the wounds (Figure S8A). Macroscopic analysis showed that wound closure was markedly attenuated in anti-miR-148b-treated wounds (Figure 6A) without influencing wound perfusion and vessel density (Figures S7C and S7D). There was not a significant improvement in wound closure detected following SMAD2 siRNA only (Figures 6A and S8B). Next, to investigate EndMT at the wound edge, wounds were stained with CD31 and FSP-1 and SLUG antibodies (Figures 6B and 6C). Confocal analysis showed that while

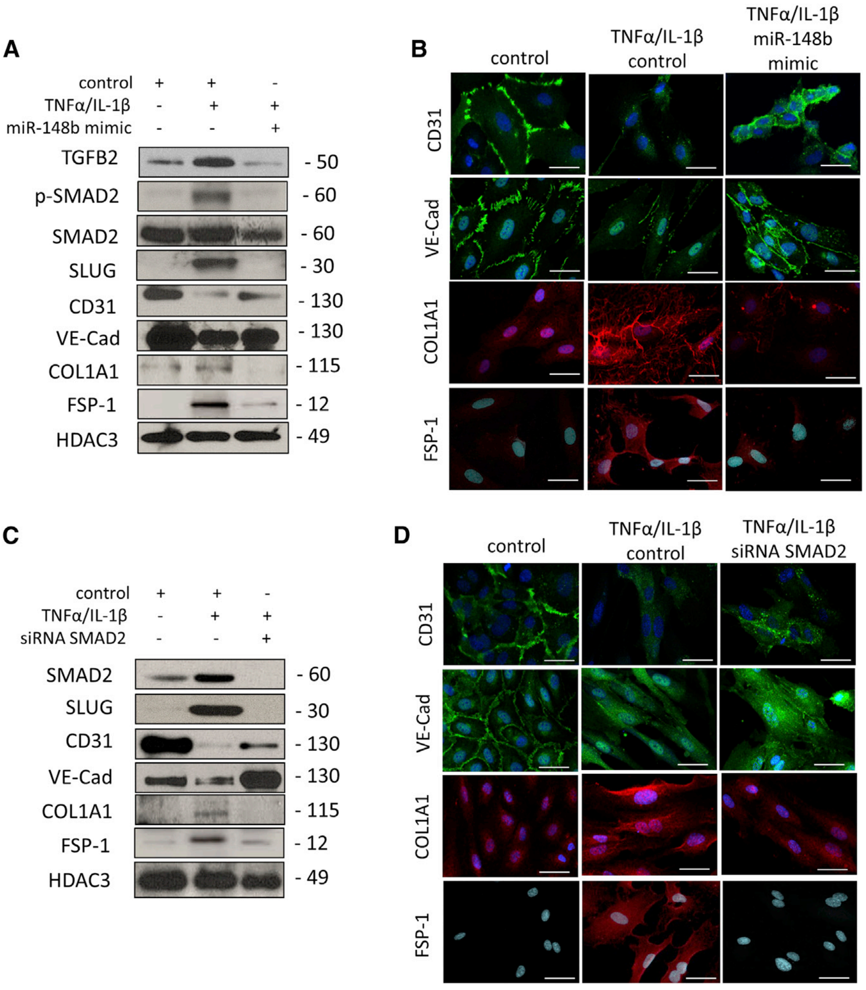

Figure 5. Overexpression of miR-148b Rescues Cytokine-Induced EndMT

HUVECs were transfected with miR-148b mimic, SMAD2 siRNA, or control and treated with TNF-α/IL-1β for 6 days. (A) Representative western blot analysis of TGFB2, SMAD2, p-SMAD2 CD31, VE-cadherin, SLUG, FSP-1, and COL1A1; HDAC3 is used as a loading control (n = 3). (B) Immunofluorescence images showing localization of CD31, VE-cadherin, COL1A1, and FSP-1 in miR-148b- or control-transfected HUVECS and/or treated with TNF-α/IL-1β; scale bars, 50 μm (magnification 400×) (n = 3). (C) Representative western blot analysis of SMAD2, CD31, VE-cadherin, SLUG, COL1A1, and FSP-1; HDAC3 is used as a loading control (n = 3). (D) Immunofluorescence images showing localization of CD31, VE-cadherin, COL1A1, and FSP-1 in SMAD2 siRNA or control-transfected HUVECS and/or treated with TNF-α/IL-1β; scale bars, 50 μm (magnification 400×) (n = 3).

control-oligonucleotide-treated wounds contained only CD31-positive vessels, anti-miR-148b-treated wounds were comprised of CD31-FSP-1 or CD31-SLUG double-positive vessels, consistent with the occurrence of EndMT (Figure 6D). Moreover, anti-miR-148b-treated wounds showed an increase in the total number of FSP-1-positive cells compared with the control oligonucleotide-treated wounds (Figure 6D), whereas staining with PicroSirius Red did not detect any increase of collagen deposition in the wounds (Figure S8E). Finally, to confirm that loss of miR-148b-mediated EndMT occurs through SMAD2 regulation, we knocked down SMAD2 by siRNA *in vivo* in the anti-miR-148b-treated wounds (Figure S8B). Knocking down SMAD2 in this model promotes wound closure (Figure 6A) without significantly promoting wound perfusion and vessel density (Figures S8C and S8D). Moreover, SMAD2 siRNA reduced the percentage of vessels double positive for CD31-FSP-1 and CD31-SLUG and decreased the total number of FSP-1-positive cells in the wounds treated with anti-miR-148b (Figure 6D). Overall, these results show that inhibition of miR-148b *in vivo* impaired wound healing and promoted EndMT on the wound edge via SMAD2.

DISCUSSION

We have comprehensively explored and been able to demonstrate for the first time that miR-148b has a crucial role in determining EC function and plasticity by regulating the TGF-β pathway. Overexpression of miR-148b enhances EC proliferation, migration, and *in vitro* angiogenesis via targeting *TGFB2* and *SMAD2*. *In vivo* delivery of miR-148b to injured skin promotes angiogenesis and accelerates wound closure. In contrast, miR-148b inhibition promotes the acquisition of a mesenchymal phenotype by ECs by unlocking the expression of *TGFB2* and *SMAD2*. This is associated with poor *in vivo* skin wound closure and activation of EndMT in dermal vessels.

One of the first indications of the involvement of miRNAs in EndMT came from Ghosh et al.,[20] who reported differential expression of a specific set of miRNAs during transition of mouse cardiac ECs to a mesenchymal phenotype. Since then, miRNAs have been linked to regulation of EndMT through different mechanisms. *In vitro* and *in vivo* studies demonstrated that decreased fibroblast growth factor (FGF) signaling leads to reduction in let-7 expression and initiation of TGF-β-dependent Endo-MT.[31] Recently, miR-20a was shown to target multiple genes in TGF-β signaling, such as ALK5, TGF-βR2, and SARA, thereby negatively regulating EndMT. The expression of miR-20a, which is normally downregulated during EndMT, is rescued by FGF2.[32] In our study, both miR-20a and let-7b are downregulated *in vivo* in the model of wound healing, whereas only miR-20a is also downregulated by cytokine treatment in HUVECs. Interestingly, further bioinformatic analysis demonstrated that the three miRNAs target a different set of genes of the TGF-β pathway with minimal (2 on 20 genes between miR-148b and let-7b) or no overlapping

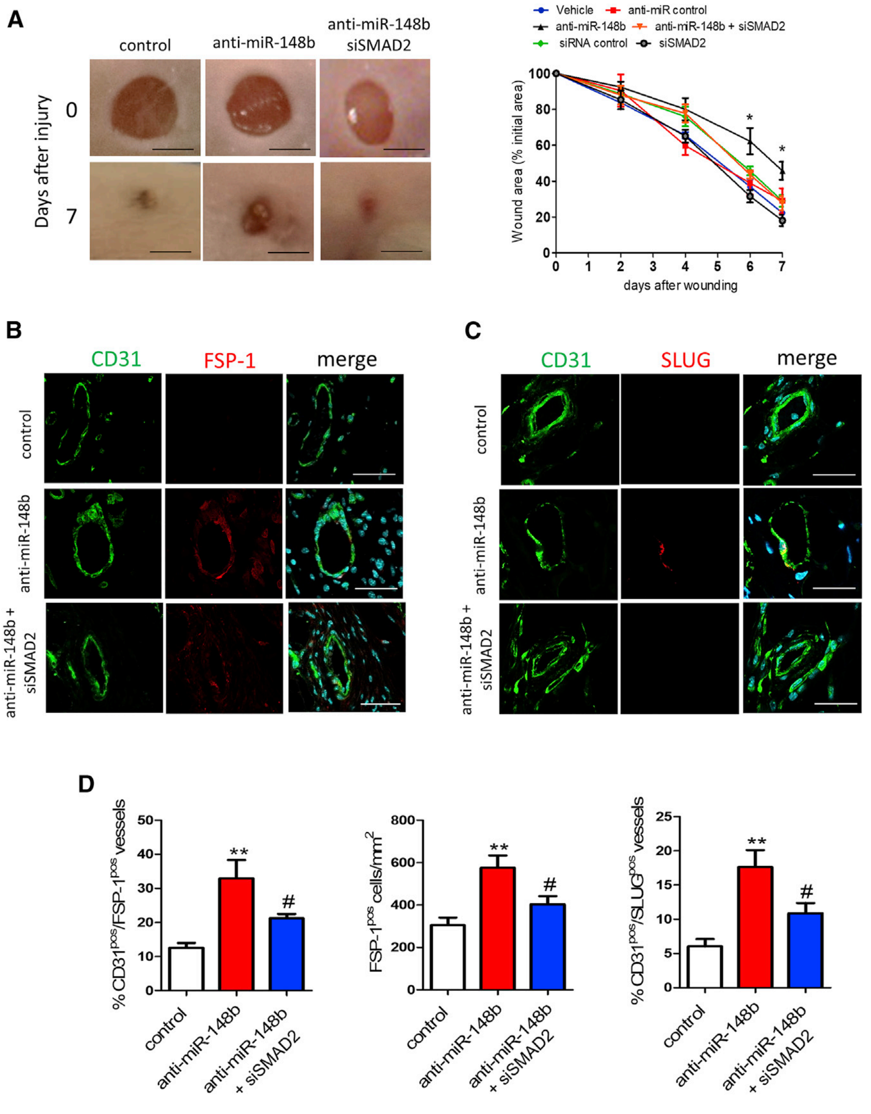

Figure 6. Inhibition of miR-148b Delays Wound Closure and Promotes EndMT in a Model of Wound Healing

Dermal wounds were treated with anti-miR control or siRNA control oligonucleotides and anti-miR-148b and/or SMAD2 siRNA for 7 days. (A) Left, representative images of treated wounds at 0 and 7 days post-wound-injury; scale bar, 5 mm. Right, level of wound closure is expressed as a percentage of wound area from the initial wound area (n = 8). (B) Immunohistochemical localization of CD31 (green) and FSP-1 (red) in the wound vessels; scale bars, 25 μm (magnification 630×) (n = 8), nuclei are stained with DAPI (blue). (C) Immunohistochemical localization of CD31 and SLUG in wound vessels; scale bars, 25 μm (magnification 630×) (n = 8), nuclei are stained with DAPI (blue). (D) Quantification of CD31/FSP-1 double-positive vessels and FSP-1-positive cells and CD31/SLUG double-positive vessels in the wounds; n = 8 per each group. Values are expressed as means ± SEM. *$p < 0.05$; **$p < 0.01$ versus anti-miR control. $^{\#}p < 0.05$ versus anti-miR-148b. Unpaired two-tailed Student's t test and one-way ANOVA statistical test followed by Bonferroni post-hoc analyses were applied.

(between miR-148b and miR-20a) targets. Therefore, miR-20a, let-7b, and miR-148b can interfere at different levels of the TGF-β pathway, permitting a possible synergic effect during skin wound healing.

The occurrence of EndMT post-natally has been associated with different types of fibrotic disease such as that in kidney[12] and heart,[11] both of which are associated with excessive collagen expression and deposition. Previous studies reported the importance of EndMT in dermal fibrosis, showing that delay in wound closure is associated with excessive collagen deposition.[33] In the present study, anti-miR-148b-mediated EndMT have a detrimental influence on skin wound closure, showing that around 30% of skin vessels are double positive for CD31 and FSP-1. This is highly important because, so transformed, these cells can be expected to secrete large amounts of collagen and other ECM proteins, which could contribute to fibrosis. In line with this, in our model we detected an increase in the recruitment of FSP-1-positive cells in wound treated with anti-miR-148b. However, analysis with PicroSirius Red did not show any accumulation of collagen in the wounds treated with anti-miR-148b at day 7 after wounding.

EndMT may lead to ECs acquiring a variety of different mesenchymal fates through different stages of differentiation.[4] The early endothelial response is characterized by a partial downregulation of endothelial markers, junction dismantling, and upregulation of some early mesenchymal markers. At later times, expression of endothelial markers further declines while more mesenchymal markers, including matrix proteins, are upregulated.[4] An abnormally increased inflammatory environment is often linked with phenotypic changes in ECs.[34] Chronic treatment of ECs with a combination of TNF-α and IL-1β over 6 days decreased the expression of miR-148b in ECs while it increased the expression and secretion of TGF-β2. In addition, the acquisition of a mesenchymal phenotype due to inflammation by dermal ECs is likely to lead to endothelial dysfunction, seen through decreased EC migration or dysregulation of inflammatory cell recruitment.[33]

Inflammation is sufficient to induce all events required for EndMT except for *de novo* acquisition of α-SMA, which is strictly dependent on TGF-β [30]. In our experiments, cytokine treatment or miR-148b inhibition increases the release of TGF-β2, as a direct target, which drives the EndMT process, perhaps partly or incompletely at least after 6 days. Consequently, EndMT appeared complete after 14 days of

TNF-α/IL-1β exposure. By this point, ECs have acquired α-SMA in addition to other markers of EndMT.

Recent reports show that inflammatory cytokines such as TNF-α and IL1-β can also trigger EndMT via binding to their receptors and by inducing nuclear factor κB (NF-κB) translocation to the nucleus, driving the expression of EndMT-specific genes.[30,35] Such inflammatory signals facilitate EndMT by increasing endogenous TGF-β expression in an NFκB-dependent manner, creating a feed-forward signaling mechanism.[36] Interestingly, treatment with the miR-148b mimics inhibited EndMT progression in cytokine-treated ECs. In agreement with this, a recent study demonstrated that pro-inflammatory molecules present in the serum of patients with Kawasaki syndrome induce EndMT through downregulation of miR-483 in ECs, whereas the overexpression of miR-483 using mimic oligonucleotides suppresses EndMT and restores EC function.[37]

In our *in vitro* model, the mechanism is based on activation of SMAD2, as demonstrated by rescue of EndMT using SMAD2 antisense. Alternatively, we have used SB43152, a specific inhibitor of ALK5, at the dose of 10 μM to inhibit SMAD2 phosphorylation and inhibit EndMT progression. However, SB43152 did not restore endothelial markers in the same manner as miR-148b mimics and SMAD2 siRNA. Discrepancy in outcomes is likely to be explained by an action of the miR-148b mimic or SMAD2 siRNA through SMAD2 mRNA degradation. In addition, another possible explanation of lack of activity of the inhibitor of ALK5 is that SMAD2 could be phosphorylated independently of ALK5 signaling by the mitotic kinase Mps1.[38]

In the *in vivo* model of wound healing, SMAD2 siRNA rescued anti-miR-148b-mediated EndMT and increased wound closure rate at day 7. However, at the same time point SMAD2 siRNA alone had no effect on wound closure rate. The discrepancy of these results could be explained by a role for other SMAD-independent pathways during wound closure and EndMT. For example, TGF-β and inflammatory cytokines have been shown to activate diverse non-SMAD parallel downstream pathways, such as extra-cellular signal-regulated kinase (ERK), c-Jun NH2-terminal kinase (JNK), p38 mitogen-activated protein (MAP) kinase.[39] Moreover, a recent study that analyzed the impact of EndMT on the skin wounds showed the existence of Notch-mediated EndMT during skin wound healing.[40]

The participation of EndMT during wound healing merits further confirmation and validation in future studies from individuals with recognized clinical pathological conditions, such as diabetic non-healing wounds. Such confirmation should lead to a change in the paradigm of the origin of cells involved in the fibrotic process. Furthermore, identification of the source of TGF-β and other active ligands that can initiate EndMT and increased understanding of the molecular mechanisms that regulate EndMT will be of great value in providing cellular targets amenable to therapeutic intervention for fibrotic disease. In our experience, the skin-wound-healing model is an excellent model to assess the potential impact of EndMT on the vessels that have formed during the wound-healing process. The excisional wounds rapidly close, with the active tissue remodelling of the wound area occurring in a reproducible way at specific time points. The use of an endothelial lineage-tracing mouse model during wound healing[40] has recently further corroborated our data and highlighted the critical role of EndMT during skin wound healing.

The TGF-β signaling pathway is considered a promising target for the treatment of many diseases, including pathological skin conditions. Most of the components of the TGF-β pathway have been targeted for drug development through various strategies. Several, such as oligonucleotide antisense, have been developed through pre-clinical to clinical trials.[41] Indeed, TGF-β antisense oligonucleotides reduced scarring and improved surgical outcome in animal models.[42] Moreover, Smad3 antisense oligonucleotides accelerated wound healing and reduced scarring in a mouse excisional wound model.[43] In this context, the benefit of miRNA-based therapy is the possibility of interfering with the regulation of multiple genes. Several miRNAs have already been used as therapeutic tools in vascular disease[17,18] to regulate different steps of the skin-wound-healing process.[44] Considering this, we have demonstrated that miR-148b mimics impact on different components of TGF-β pathway, thus amplifying its therapeutic potential in a model of skin wound healing.

An important factor that we have considered in our *in vivo* experiments is also at which stage of the wound-healing process treatment with miR-148b mimics could be beneficial. Several studies have previously reported that reducing TGF-β pathway at later stages during skin wound healing improved scarring outcome.[45] Based on these observations, skin wounds in the present study were treated from 3 days after wounding, when expression of miR-148b decreased, thus avoiding early inhibition of TGF-β signaling during the inflammatory phase, when it is required to start the healing process.[46]

In summary, the data presented here identify miR-148b as a novel regulator of TGF-β signaling and EC plasticity and a potential therapeutic target for promotion of tissue repair.

MATERIALS AND METHODS

Cells and Cell Culture

HUVECs (Lonza) were grown in endothelial growth medium-2 (EGM-2) (comprising endothelial basal medium-2 [EBM-2] with growth factors and other supplements) with 2% fetal bovine serum (FBS). Confluent HUVECs were treated with 10 ng/mL TNF-α, IL-1β, and TGF-β2 (PeproTech); medium and cytokines were changed every 2 days. SB431542 (Tocris) has been used at the dose of 10 μM. HEK293T cells (ATCC, CRL-11268) were cultured in DMEM with 10% FBS (Life Technologies).

miR-148b Target Analysis

Computational prediction of miR-148b target genes was done using published algorithm TargetScan (http://www.targetscan.org). miR-path v.3 (http://diana.imis.athena-innovation.gr/DianaTools/index.php?r=mirpath) was used to perform gene set enriched analysis of

miR-148b target genes. The prediction is based on TargetScan parameters such as Context score and Conservation score. The Context score for a specific site is the sum of the contribution of 14 features of the 3′ UTR structure;[47] the Conservation score is the probability of conserved targeting.[48] TargetScan considers matches to human 3′ UTRs and their orthologs, as defined by UCSC whole-genome alignments. Lowest Context score shows a more favorable binding. Higher conservation score represents a broad conservation of the binding sites between the species.

RNA Extraction and Quantitative Real-Time Analysis

Total RNA was extracted using an miReasy kit (QIAGEN). Real-time quantification to measure miRNA was performed with the TaqMan miRNA reverse transcription kit and miRNA assay (Life Technologies) using Lightcycler 480 (Roche). miRNA expression was normalized to the U6 small nucleolar RNA (snRU6). For mRNA analysis, cDNA was amplified by quantitative real-time PCR (real-time qPCR) and normalized to 18S ribosomal RNA. Each reaction was performed in triplicate. Quantification was performed by the $2^{-\Delta\Delta Ct}$ method.[49] Real-time qPCR was used to measure the expression of miR-148b (miR-148b-3p Thermo Fisher Scientific, cat. #4426961), snRU6 (Thermo Fisher Scientific, cat. #4331182), TGF-β2, SMAD2, CD31, VE-cadherin, COL1A1, α-SMA, SLUG, VIM, SNAIL, TWIST, ZEB1/2, and 18S rRNA. Pre-optimized primers were obtained from Sigma (KiCqStart Primers).

Cell Transfection, Transduction, and Functional Assays

Lipofectamine RNAiMAX (Thermo Fisher Scientific) was used to transfect HUVECs with miR-148b mimic, anti-miR-148b, and mimic or anti-miR control (25 nM and 75 mM, respectively, final concentration) or with siRNA targeting SMAD2 (20 nM final concentration), according to the manufacturer's instructions. jetPEI-HUVECs (Polyplus) was used to transfect HUVECs with DNA plasmids. The following functional assays were performed: BrdU incorporation assay using Cell Proliferation colorimetric assay (Roche); Matrigel assay with HUVECs was performed as previously described using BD Matrigel Basement Membrane Matrix (BD Biosciences).[50]

ECIS Assays: Migration Assay and Plasma Membrane Capacitance Analysis

Confluent HUVECs were transfected with controls or miR-148b mimic or anti-miR-148b and plated on the electric cell-substrate impedance sensing (ECIS) chip array (8W1E or 8W10E). The migration speed was calculated in μm/hr, capacitance of the plasma membrane is capacitance (μF/cm^2) as reported in Giaever and Keese.[29]

Western Blot Analyses

Proteins were extracted from cultured cells or muscles by using ice-cold buffer A (50 mM HEPES, 150 mM NaCl, 1 mM EDTA, 1 mM EGTA, 25 mM sodium fluoride [NaF], 5 mM sodium pyrophosphate [$Na_4P_2O_7$], 1% Triton, 1% NP40, 1 mM sodium orthovanadate [Na3VO4], 0.25% sodium deoxycholate, 0.5 mM Na-orthovanadate, 1 mM benzamidine, 0.1 mM phenylmethylsulfonyl fluoride). Protein concentration was determined using the Bio-Rad Protein Assay Reagent (Bio-Rad, UK). Detection of proteins by western blot analyses was done following separation of whole-cell extracts (20 μg) on SDS-polyacrylamide gels. Proteins were transferred to nitrocellulose membranes and probed with the following antibodies: TGF-β2 (Abcam, ab36495; 1:1,000), SMAD2 (Santa Cruz Biotechnology, Santa Cruz, CA, USA, #3102, 1:1,000), p-SMAD2 (465/467) (Cell Signaling, #3108; 1:1,000); CD31 (Abcam, 1:1,000), VE-cadherin (Abcam, ab33168; 1:1,000), COL1A1 (Abcam, ab138492; 1:200), FSP-1 (S100A4; Abcam, ab41532; 1:1,000), SLUG (Abcam, ab27568; 1:1000), β-actin (Abcam, ab16039; 1:4,000), HDAC3 (GeneTex GTX113303; 1:1,000), and GAPDH (GeneTex, GT239; 1:1,000) (both used as loading control). For detection, we used secondary antibodies conjugated to horseradish peroxidase, which were rabbit anti-mouse (Abcam, ab97046; 1:5,000) and goat anti-rabbit (Abcam, ab6721; 1:10,000). Detection was developed by enhanced chemiluminescence reaction (ECL) (Immunoblot, Millipore, 23225).

Immunofluorescence

Cells were fixed using 4% paraformaldehyde in PBS at room temperature for 15 min. For intracellular staining, fixed cells were permeabilized using 0.5% Triton X-100 in PBS (Sigma-Aldrich) or 0.1% Saponin in PBS at room temperature for 10 min. The blocking of specific antibody activity was performed using 3% BSA in PBS for 1 hr. Samples were incubated with antibodies to CD31 (Abcam, ab28364; 1:50), VE-cadherin (Abcam, ab33168; 1:100), FSP-1 (Abcam, ab41532; 1:200) and COL1A1 (Abcam, ab138492; 1:100) in PBS containing 1% BSA at 4°C overnight. Samples were washed extensively with PBS and incubated with Alexa Fluor 488-conjugated antibodies to rabbit immunoglobulin G (IgG) (Life Technologies, Carlsbad, CA, #A11070) and Alexa Fluor 594-conjugated antibodies to rabbit IgG (Life Technologies, Carlsbad, CA, #A11072) in DAPI with PBS with 1% BSA at room temperature for 1 hr. Image analysis was performed on Zeiss LSM 780 confocal microscope.

Luciferase Assay

Luciferase assay has been performed as previously described.[50] TGFB2 3′ UTR and SMAD2 3′ UTR vectors were cloned in pMIR-Reporter (Life Technologies). Primers are used for the cloning are as follows: TGF-β2 3′ UTR, forward 5′-ATAAAGCTTATTTGCCA CATCATTGCAGA-3′, reverse 5′-ATAACTAGTGGGAATAAAAA GACGGCACA-3′; SMAD2 3′ UTR, forward 5′-ATAAAGCTTT GATCCAGCTAAGGTAACTGATGTT-3′; reverse 5′-ATAACTAG TTGGTAAACAACTCAAATGGCTTTC-3′. Primers for 3′ UTR mutation are as follows: TGFB2, forward 5′-ATAAAGCTTATTTG CCACATCATTGCAGA-3′ and reverse 5′-ATAACTAGTCCTATC TGAGAGGAAAATGTCTGC-3′; SMAD2, forward 5′-ATAAAGC TTTGATCCAGCTAAGGTAACTGATGTT-3′ and reverse 5′-ATA ACTAGTGGACTTCCAGAGGGAAACAA-3′. Luciferase constructs were transfected into HEK293T cells or HUVECs together with miR-148b mimics or p-SV-β-gal control vector. Cells were cultured for 48 hr and assayed with the Luciferase and β-Galactosidase Reporter Assay Systems (Promega). Luciferase values were normalized to protein concentration and β-galactosidase activity.

Animal Experiments

All experiments involving mice were performed in accordance with the guidance and the operation of Animals (Scientific Procedures) Act 1986 and prior approval of the UK Home Office and the University of Edinburgh Animal Welfare and Ethical Review Board. The skin-wound-healing protocol was performed as described previously.[51–53] CD-1 female mice (7 to 9 weeks old) (n = 8 per group) were randomly assigned to a treatment group and anaesthetized with isofluorane. Two full-thickness excisional wounds were made to the shaved dorsal skin. Full-thickness excisional wounds were made in a midline skin fold using a sterile, disposable 5-mm biopsy punch (Kai Industries), resulting in generation of one wound on each side of the midline. Control oligonucleotides (scramble sequence), miR-148b mimic, Cy3-labeled miR-148b mimic, or anti-miR-148b or SMAD2 siRNA (1 μg/wound) were delivered topically by pipette into the wound cavity (10 μL in a vehicle of 30% Pluronic F-127 gel [that is, liquid at 4°C but solidifies at body temperature]; Sigma-Aldrich). Mimics were initially delivered after 3 days, whereas anti-miR and siRNA were delivered immediately after wounding. The treatment was repeated every second day for 7 days. Wounds were photographed using an Olympus camera on days 0, 2, 4, 6, and 7 after wounding. Wound areas were calculated as previously described.[51,52] In brief, wound diameter was measured using a Vernier caliper, and wound area was calculated using a standard formula for the area of an ellipse (semi-major diameter × semi-minor diameter × Pi). The superficial blood flow of the wounds was sequentially analyzed by color laser Doppler (Color Laser Doppler, Moor, UK), and the ratio of blood flow between the mimic or anti-miR or siRNA-treated and the control-treated wounds was calculated. Wound size and Doppler analysis were performed in blinded fashion.

Histology and Image Analysis

Wounds were harvested, prepared, and analyzed as previously described.[51–53] At days 5 or 7 post-wounding, wounds were harvested and fixed in 10% buffered formalin (16 hr at 4°C, Sigma) for embedding in paraffin. Sections were deparaffinized, rehydrated, and stained with rat anti-mouse polyclonal CD31 (Abcam, ab28364; 1:50), α-SMA (Sigma, A2547; 1:100), rabbit anti-mouse FSP-1 (Abcam, ab41532; 1:50), and SLUG (Abcam, ab28364; 1:200); all antibodies were used overnight at 4°C. Stained slides were photographed and analyzed in a blinded fashion using a Zeiss LSM 780 Confocal Microscope (Zeiss). Vascular density in wounds was counted after immunostaining for CD31 and α-SMA. Ten fields per section/animal (n = 8 animal; 400× magnification) were randomly examined and averaged to analyze the number of CD31-positive blood vessels in the wound edges. Image analysis was performed using ImageJ software as previously reported.[51] Vascular density is expressed per square millimeter. EndMT of ECs was assessed by FSP-1 and SLUG immunostaining combined with CD31 staining. CD31-FSP-1 and CD31-SLUG double-positive vessels are expressed as percentage of positive vessels. For analysis of fibrosis, slides were stained with PicroSirius Red (PSR) then photographed and analyzed in a blinded fashion using a light microscope (Axioscope 2; Zeiss). Six images per slide were captured at 400× magnification for PSR histological staining. Color deconvolution in ImageJ software was used to evaluate the percentage of red staining, which indicates the presence of collagen fibers in the tissue. The area of collagen was measured as the percentage of total pixels in each image, using the Threshold option in ImageJ software.

Statistical Analysis

Comparisons between two different conditions were assessed using two-tailed Student's t test. Differences among groups of more than two were tested using one-way ANOVA, followed by Bonferroni post-hoc analyses as appropriate. Data are expressed as mean ± SEM of three independent experiments, each performed in triplicate or quintuplicate, as reported in the figure legends. A p value < 0.05 was considered statistically significant. Analyses were performed using GraphPad Prism v5.0.

SUPPLEMENTAL INFORMATION

Supplemental Information includes eight figures and can be found with this article online at https://doi.org/10.1016/j.ymthe.2018.05.002.

AUTHOR CONTRIBUTIONS

V.M. acquired and analyzed the data and drafted the manuscript; M.M., T.M., A.A.H.Z., A.M., L.R., B.C., G.A.G., and E.P. participated in acquiring and analyzing the data and revised the manuscript; A.C. designed the study and drafted the manuscript.

CONFLICTS OF INTEREST

The authors declare no competing interest.

ACKNOWLEDGMENTS

V.M. is sponsored by a University of Edinburgh Chancellor's Fellow studentship and A.C. is a British Heart Foundation (BHF) Intermediate Research Fellow and a University of Edinburgh Chancellor's Fellow. This study was supported by grants from the British Heart Foundation (BHF) (FS/11/52/29018 and PG/16/58/32275 to A.C. and FS/14/7/30574 to A.M.). B.C. is supported by the Edinburgh BHF PhD Program, A.A.H.Z. is supported by the Intermediate Fellowship awarded by the Oxford British Heart Foundation (BHF) Centre of Research Excellence (RE/08/004/23915), and T.M. is supported by BHF Career Re-entry Fellow (FS/16/38/32351). A.C. acknowledges the Edinburgh BHF Research Excellence Award.

REFERENCES

1. Goumans, M.J., and Ten Dijke, P. (2018). TGF-beta signaling in control of cardiovascular function. Cold Spring Harb. Perspect. Biol. *10*, a022210.
2. van Meeteren, L.A., and ten Dijke, P. (2012). Regulation of endothelial cell plasticity by TGF-β. Cell Tissue Res. *347*, 177–186.
3. Akhurst, R.J. (2012). The paradoxical TGF-β vasculopathies. Nat. Genet. *44*, 838–839.
4. Dejana, E., Hirschi, K.K., and Simons, M. (2017). The molecular basis of endothelial cell plasticity. Nat. Commun. *8*, 14361.
5. Piera-Velazquez, S., Mendoza, F.A., and Jimenez, S.A. (2016). Endothelial to mesenchymal transition (EndoMT) in the pathogenesis of human fibrotic diseases. J. Clin. Med. *5*, e45.

6. Kovacic, J.C., Mercader, N., Torres, M., Boehm, M., and Fuster, V. (2012). Epithelial-to-mesenchymal and endothelial-to-mesenchymal transition: from cardiovascular development to disease. Circulation *125*, 1795–1808.

7. Piera-Velazquez, S., and Jimenez, S.A. (2012). Molecular mechanisms of endothelial to mesenchymal cell transition (EndoMT) in experimentally induced fibrotic diseases. Fibrogenesis Tissue Repair *5* (*Suppl 1*), S7.

8. Arciniegas, E., Neves, C.Y., Carrillo, L.M., Zambrano, E.A., and Ramírez, R. (2005). Endothelial-mesenchymal transition occurs during embryonic pulmonary artery development. Endothelium *12*, 193–200.

9. Armstrong, E.J., and Bischoff, J. (2004). Heart valve development: endothelial cell signaling and differentiation. Circ. Res. *95*, 459–470.

10. Zeisberg, E.M., Potenta, S., Xie, L., Zeisberg, M., and Kalluri, R. (2007). Discovery of endothelial to mesenchymal transition as a source for carcinoma-associated fibroblasts. Cancer Res. *67*, 10123–10128.

11. Zeisberg, E.M., Tarnavski, O., Zeisberg, M., Dorfman, A.L., McMullen, J.R., Gustafsson, E., Chandraker, A., Yuan, X., Pu, W.T., Roberts, A.B., et al. (2007). Endothelial-to-mesenchymal transition contributes to cardiac fibrosis. Nat. Med. *13*, 952–961.

12. Zeisberg, E.M., Potenta, S.E., Sugimoto, H., Zeisberg, M., and Kalluri, R. (2008). Fibroblasts in kidney fibrosis emerge via endothelial-to-mesenchymal transition. J. Am. Soc. Nephrol. *19*, 2282–2287.

13. Cooley, B.C., Nevado, J., Mellad, J., Yang, D., Hilaire, C.S., Negro, A., Fang, F., Chen, G., San, H., Walts, A.D., et al. (2014). TGF-β signaling mediates endothelial-to-mesenchymal transition (EndMT) during vein graft remodeling. Sci. Transl. Med. *6*, 227ra34.

14. Evrard, S.M., Lecce, L., Michelis, K.C., Nomura-Kitabayashi, A., Pandey, G., Purushothaman, K.R., d'Escamard, V., Li, J.R., Hadri, L., Fujitani, K., et al. (2016). Endothelial to mesenchymal transition is common in atherosclerotic lesions and is associated with plaque instability. Nat. Commun. *7*, 11853.

15. Ha, M., and Kim, V.N. (2014). Regulation of microRNA biogenesis. Nat. Rev. Mol. Cell Biol. *15*, 509–524.

16. Li, Z., and Rana, T.M. (2014). Therapeutic targeting of microRNAs: current status and future challenges. Nat. Rev. Drug Discov. *13*, 622–638.

17. Caporali, A., and Emanueli, C. (2011). MicroRNA regulation in angiogenesis. Vascul. Pharmacol. *55*, 79–86.

18. Caporali, A., and Emanueli, C. (2012). MicroRNAs in postischemic vascular repair. Cardiol. Res. Pract. *2012*, 486702.

19. Kurakula, K., Goumans, M.J., and Ten Dijke, P. (2015). Regulatory RNAs controlling vascular (dys)function by affecting TGF-β family signalling. EXCLI J. *14*, 832–850.

20. Ghosh, A.K., Nagpal, V., Covington, J.W., Michaels, M.A., and Vaughan, D.E. (2012). Molecular basis of cardiac endothelial-to-mesenchymal transition (EndMT): differential expression of microRNAs during EndMT. Cell. Signal. *24*, 1031–1036.

21. Chen, Y., Song, Y.X., and Wang, Z.N. (2013). The microRNA-148/152 family: multi-faceted players. Mol. Cancer *12*, 43.

22. Serino, G., Sallustio, F., Cox, S.N., Pesce, F., and Schena, F.P. (2012). Abnormal miR-148b expression promotes aberrant glycosylation of IgA1 in IgA nephropathy. J. Am. Soc. Nephrol. *23*, 814–824.

23. Bidzhekov, K., Gan, L., Denecke, B., Rostalsky, A., Hristov, M., Koeppel, T.A., Zernecke, A., and Weber, C. (2012). microRNA expression signatures and parallels between monocyte subsets and atherosclerotic plaque in humans. Thromb. Haemost. *107*, 619–625.

24. Vlachos, I.S., Zagganas, K., Paraskevopoulou, M.D., Georgakilas, G., Karagkouni, D., Vergoulis, T., Dalamagas, T., and Hatzigeorgiou, A.G. (2015). DIANA-miRPath v3.0: deciphering microRNA function with experimental support. Nucleic Acids Res. *43* (W1), W460–W466.

25. Grimson, A., Farh, K.K., Johnston, W.K., Garrett-Engele, P., Lim, L.P., and Bartel, D.P. (2007). MicroRNA targeting specificity in mammals: determinants beyond seed pairing. Mol. Cell *27*, 91–105.

26. Song, H., Wang, Q., Wen, J., Liu, S., Gao, X., Cheng, J., and Zhang, D. (2012). ACVR1, a therapeutic target of fibrodysplasia ossificans progressiva, is negatively regulated by miR-148a. Int. J. Mol. Sci. *13*, 2063–2077.

27. Zhang, J., Ying, Z.Z., Tang, Z.L., Long, L.Q., and Li, K. (2012). MicroRNA-148a promotes myogenic differentiation by targeting the ROCK1 gene. J. Biol. Chem. *287*, 21093–21101.

28. Simons, M., Alitalo, K., Annex, B.H., Augustin, H.G., Beam, C., Berk, B.C., Byzova, T., Carmeliet, P., Chilian, W., Cooke, J.P., et al.; American Heart Association Council on Basic Cardiovascular Sciences and Council on Cardiovascular Surgery and Anesthesia (2015). State-of-the-art methods for evaluation of angiogenesis and tissue vascularization: a scientific statement from the American Heart Association. Circ. Res. *116*, e99–e132.

29. Giaever, I., and Keese, C.R. (1991). Micromotion of mammalian cells measured electrically. Proc. Natl. Acad. Sci. USA *88*, 7896–7900.

30. Rieder, F., Kessler, S.P., West, G.A., Bhilocha, S., de la Motte, C., Sadler, T.M., Gopalan, B., Stylianou, E., and Fiocchi, C. (2011). Inflammation-induced endothelial-to-mesenchymal transition: a novel mechanism of intestinal fibrosis. Am. J. Pathol. *179*, 2660–2673.

31. Chen, P.Y., Qin, L., Barnes, C., Charisse, K., Yi, T., Zhang, X., Ali, R., Medina, P.P., Yu, J., Slack, F.J., et al. (2012). FGF regulates TGF-β signaling and endothelial-to-mesenchymal transition via control of let-7 miRNA expression. Cell Rep. *2*, 1684–1696.

32. Correia, A.C., Moonen, J.R., Brinker, M.G., and Krenning, G. (2016). FGF2 inhibits endothelial-mesenchymal transition through microRNA-20a-mediated repression of canonical TGF-β signaling. J. Cell Sci. *129*, 569–579.

33. Manetti, M., Romano, E., Rosa, I., Guiducci, S., Bellando-Randone, S., De Paulis, A., Ibba-Manneschi, L., and Matucci-Cerinic, M. (2017). Endothelial-to-mesenchymal transition contributes to endothelial dysfunction and dermal fibrosis in systemic sclerosis. Ann. Rheum. Dis. *76*, 924–934.

34. Romero, L.I., Zhang, D.N., Herron, G.S., and Karasek, M.A. (1997). Interleukin-1 induces major phenotypic changes in human skin microvascular endothelial cells. J. Cell. Physiol. *173*, 84–92.

35. Mahler, G.J., Farrar, E.J., and Butcher, J.T. (2013). Inflammatory cytokines promote mesenchymal transformation in embryonic and adult valve endothelial cells. Arterioscler. Thromb. Vasc. Biol. *33*, 121–130.

36. Liu, R.M., and Gaston Pravia, K.A. (2010). Oxidative stress and glutathione in TGF-beta-mediated fibrogenesis. Free Radic. Biol. Med. *48*, 1–15.

37. He, M., Chen, Z., Martin, M., Zhang, J., Sangwung, P., Woo, B., Tremoulet, A.H., Shimizu, C., Jain, M.K., Burns, J.C., and Shyy, J.Y. (2017). miR-483 targeting of CTGF suppresses endothelial-to-mesenchymal transition: therapeutic implications in Kawasaki disease. Circ. Res. *120*, 354–365.

38. Zhu, S., Wang, W., Clarke, D.C., and Liu, X. (2007). Activation of Mps1 promotes transforming growth factor-beta-independent Smad signaling. J. Biol. Chem. *282*, 18327–18338.

39. Derynck, R., and Zhang, Y.E. (2003). Smad-dependent and Smad-independent pathways in TGF-beta family signalling. Nature *425*, 577–584.

40. Patel, J, Baz, B, Wong, HY, Lee, JS, and Khosrotehrani, K (2017). Accelerated endothelial to mesenchymal transition increased fibrosis via deleting Notch signalling in wound vasculature.

41. Akhurst, R.J., and Hata, A. (2012). Targeting the TGFβ signalling pathway in disease. Nat. Rev. Drug Discov. *11*, 790–811.

42. Choi, B.M., Kwak, H.J., Jun, C.D., Park, S.D., Kim, K.Y., Kim, H.R., and Chung, H.T. (1996). Control of scarring in adult wounds using antisense transforming growth factor-beta 1 oligodeoxynucleotides. Immunol. Cell Biol. *74*, 144–150.

43. Cordeiro, M.F., Mead, A., Ali, R.R., Alexander, R.A., Murray, S., Chen, C., York-Defalco, C., Dean, N.M., Schultz, G.S., and Khaw, P.T. (2003). Novel antisense oligonucleotides targeting TGF-beta inhibit in vivo scarring and improve surgical outcome. Gene Ther. *10*, 59–71.

44. Mulholland, E.J., Dunne, N., and McCarthy, H.O. (2017). MicroRNA as therapeutic targets for chronic wound healing. Mol. Ther. Nucleic Acids *8*, 46–55.

45. Finnson, K.W., McLean, S., Di Guglielmo, G.M., and Philip, A. (2013). Dynamics of transforming growth factor beta signaling in wound healing and scarring. Adv. Wound Care (New Rochelle) *2*, 195–214.

46. Finnson, K.W., Arany, P.R., and Philip, A. (2013). Transforming growth factor beta signaling in cutaneous wound healing: lessons learned from animal studies. Adv. Wound Care (New Rochelle) *2*, 225–237.

47. Agarwal, V., Bell, G.W., Nam, J.W., and Bartel, D.P. (2015). Predicting effective microRNA target sites in mammalian mRNAs. eLife *4*, eLife.05005.

48. Friedman, R.C., Farh, K.K., Burge, C.B., and Bartel, D.P. (2009). Most mammalian mRNAs are conserved targets of microRNAs. Genome Res. *19*, 92–105.

49. Schmittgen, T.D., and Livak, K.J. (2008). Analyzing real-time PCR data by the comparative C(T) method. Nat. Protoc. *3*, 1101–1108.

50. Caporali, A., Meloni, M., Völlenkle, C., Bonci, D., Sala-Newby, G.B., Addis, R., Spinetti, G., Losa, S., Masson, R., Baker, A.H., et al. (2011). Deregulation of microRNA-503 contributes to diabetes mellitus-induced impairment of endothelial function and reparative angiogenesis after limb ischemia. Circulation *123*, 282–291.

51. Al Haj Zen, A., Nawrot, D.A., Howarth, A., Caporali, A., Ebner, D., Vernet, A., Schneider, J.E., and Bhattacharya, S. (2016). The retinoid agonist tazarotene promotes angiogenesis and wound healing. Mol. Ther. *24*, 1745–1759.

52. Caporali, A., Meloni, M., Miller, A.M., Vierlinger, K., Cardinali, A., Spinetti, G., Nailor, A., Faglia, E., Losa, S., Gotti, A., et al. (2012). Soluble ST2 is regulated by p75 neurotrophin receptor and predicts mortality in diabetic patients with critical limb ischemia. Arterioscler. Thromb. Vasc. Biol. *32*, e149–e160.

53. Barcelos, L.S., Duplaa, C., Kränkel, N., Graiani, G., Invernici, G., Katare, R., Siragusa, M., Meloni, M., Campesi, I., Monica, M., et al. (2009). Human $CD133^+$ progenitor cells promote the healing of diabetic ischemic ulcers by paracrine stimulation of angiogenesis and activation of Wnt signaling. Circ. Res. *104*, 1095–1102.

YMTHE, Volume *26*

Supplemental Information

MicroRNA-148b Targets the TGF-β Pathway to Regulate Angiogenesis and Endothelial-to-Mesenchymal Transition during Skin Wound Healing

Vladislav Miscianinov, Andrea Martello, Lorraine Rose, Elisa Parish, Ben Cathcart, Tijana Mitić, Gillian A. Gray, Marco Meloni, Ayman Al Haj Zen, and Andrea Caporali

A

#	Gene Name	Context Score	Conservation Score
1.	ROCK1	-0.226	0.963
2.	SMAD2	0.030	0.762
3.	INHBB	-0.375	0.903
4.	SMURF2	-0.267	0.318
5.	ACVR1	-0.237	0.634
6.	ACVR2B	0.008	0.778
7.	TGFB2	-0.279	0.885
8.	LTBP1	-0.210	0.557
9.	NOG	-0.292	0.501

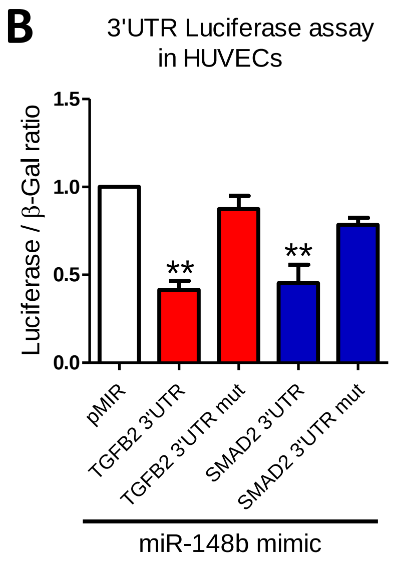

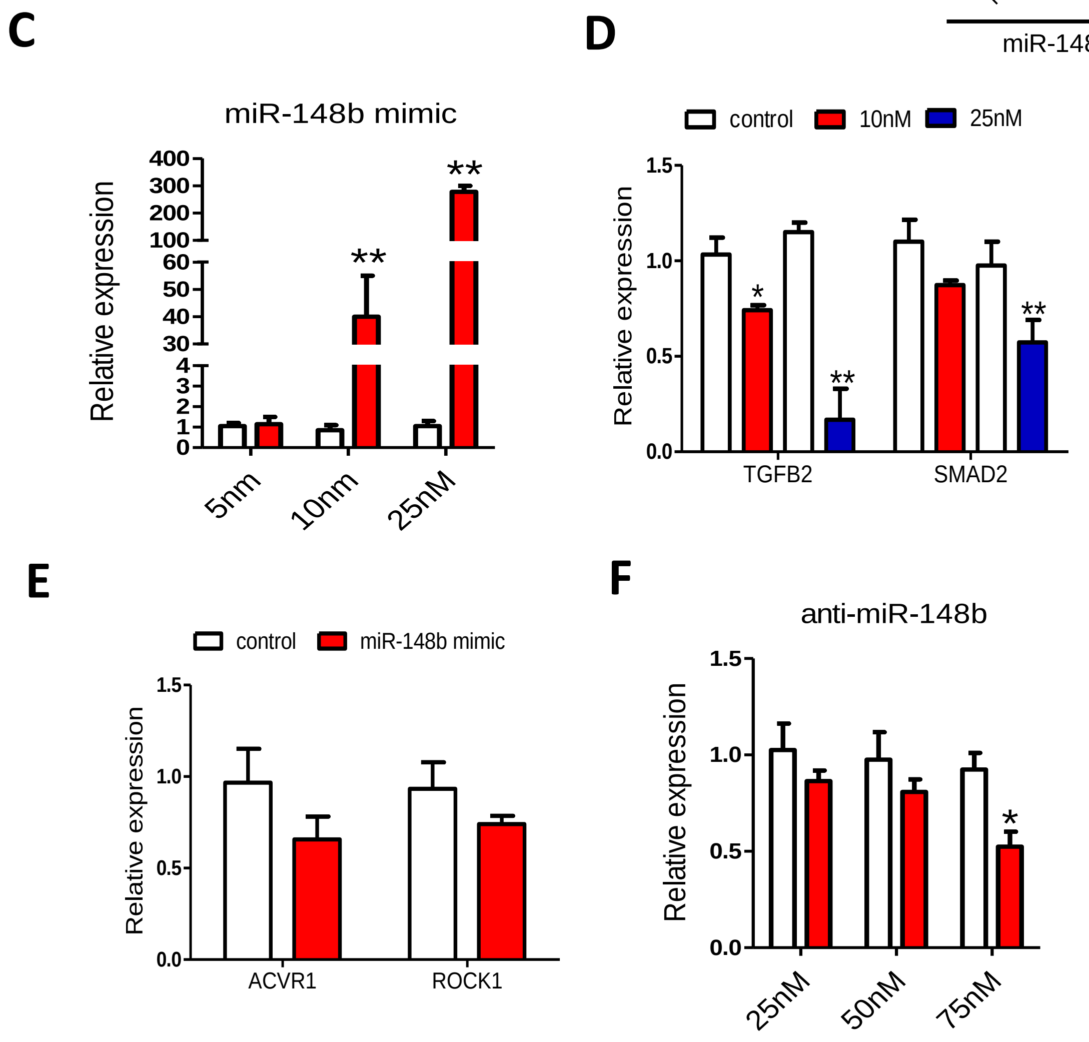

Figure S1. miR-148b *in silico* targets analysis and miR-148b expression level

A. Analysis of miR-148b target genes using miRpath software. Table shows Context Score and Conservation score from Targetscan. **B.** Luciferase activity at 48h post-co-transfection of HUVECs cells with both miR-148b and the following plasmids: 3'-UTR-TGFB2. 3'-UTR-SMAD2 and pMIR as empty plasmid (n=5); **C.** MiR-148b expression levels after miR mimic transfection (n=5); **D.** TGFB2 and SMAD2 expression after different doses of miR-148b mimic transfection (n=5); **E.** ACVR1 and ROCK1 expression miR-148b mimic transfection (n=5); **F.** MiR-148b expression levels after anti-miR-148b transfection (n=5);. Values are means±SEM. *P<0.05; **P<0.01 vs control. Unpaired two-tailed Student's t-test was applied.

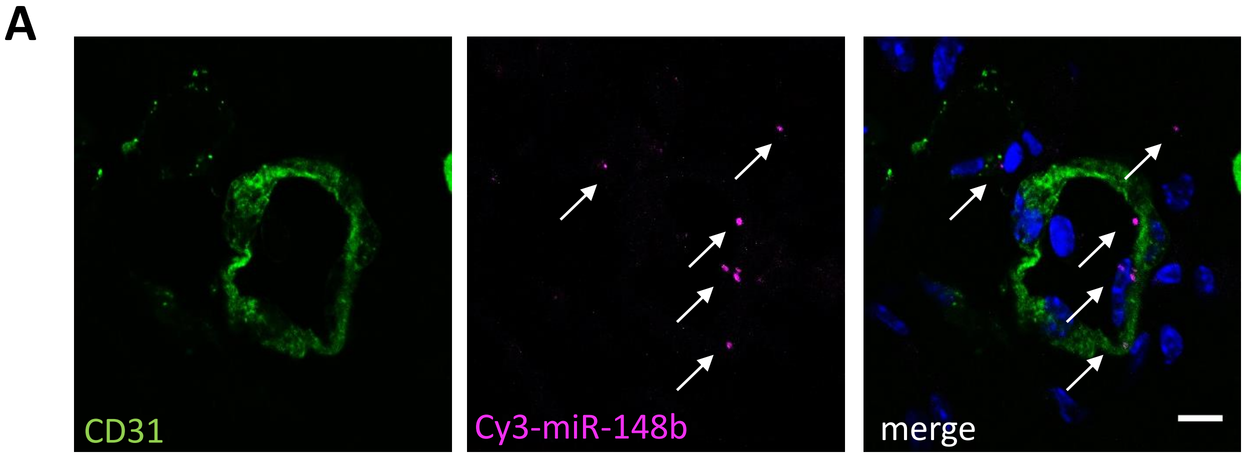

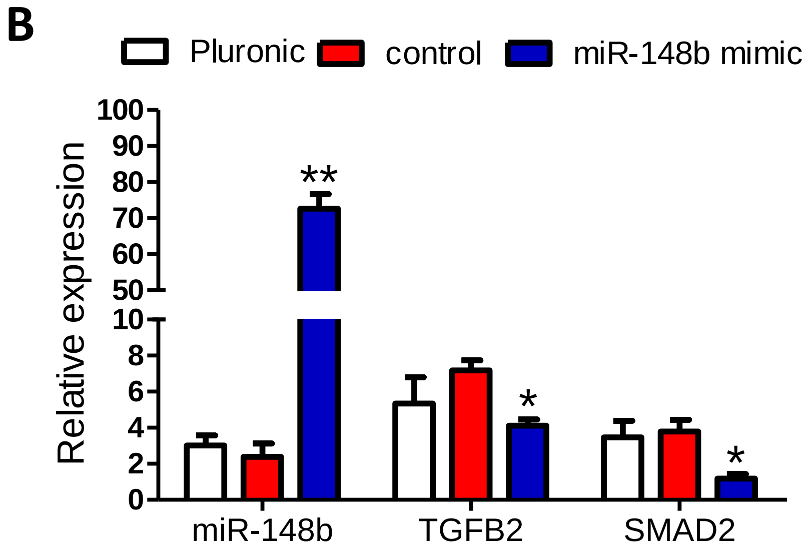

Figure S2: Delivery of miR-148b mimic *in vivo*

A. Representative images of the localization of Cy3 labelled miR-148b (pink dots) in skin wounds. Vessels are stained for CD31 (green), scale bar=25µm (magnification 1000x); **B.** Relative gene expression of miR-148b, *TGFB2* and *SMAD2* in dermal wound after delivering of miR-148b mimic or control oligonucleotides at 7 days (n=5)**;** Values are means±SEM. *P<0.05; **P<0.01 vs control or not-wounded skin. Unpaired two-tailed Student's t-test was applied.

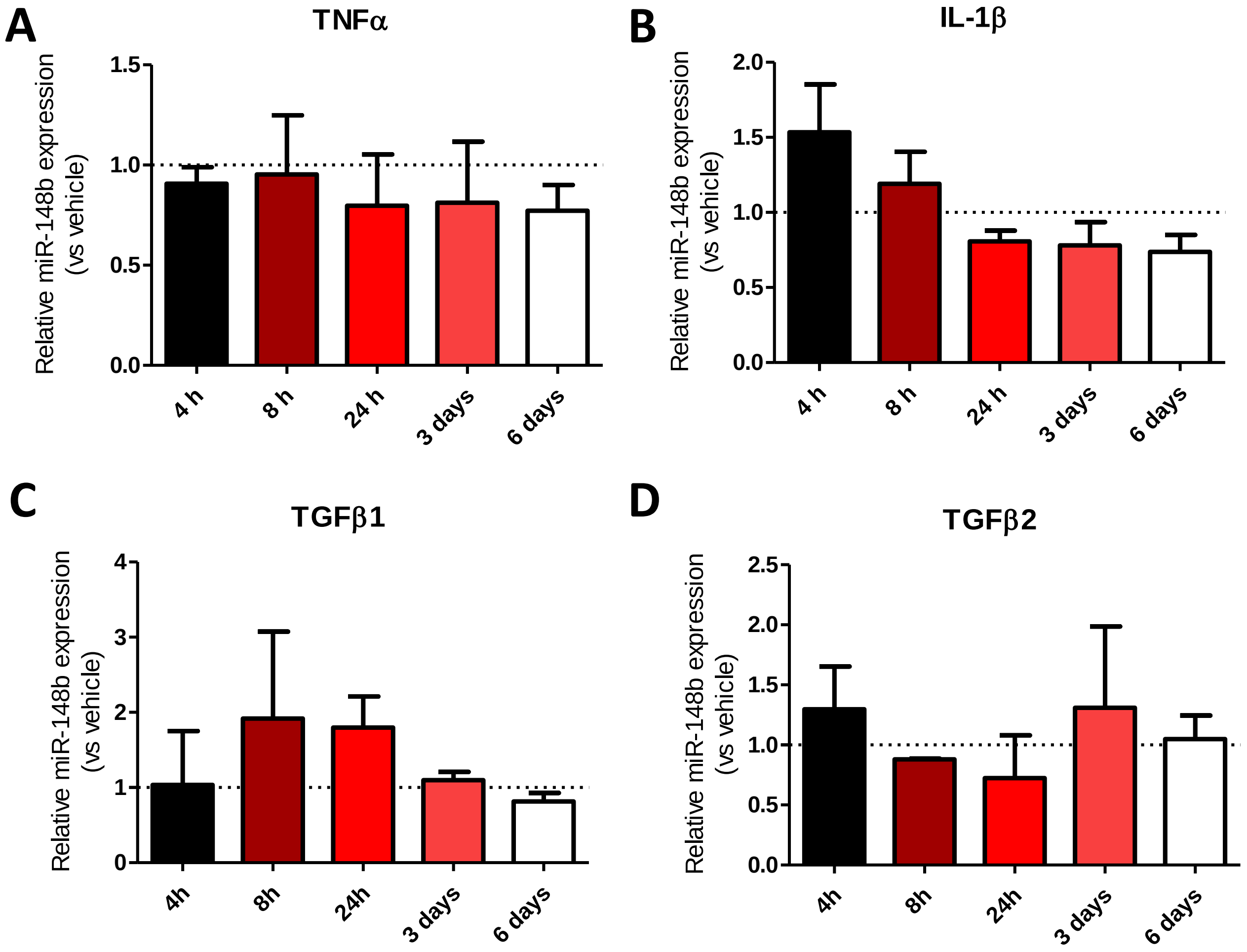

Figure S3: Regulation of miR-148b expression *in vitro*. Relative miR-148b expression after treatment of HUVECs with **A.** TNF-α; **B.** IL-1β; **C.** TGFβ1; **D.** TGFβ2; the dotted line represents relative miR-148b expression in the untreated control HUVEC (n=3); Values are means±SEM.

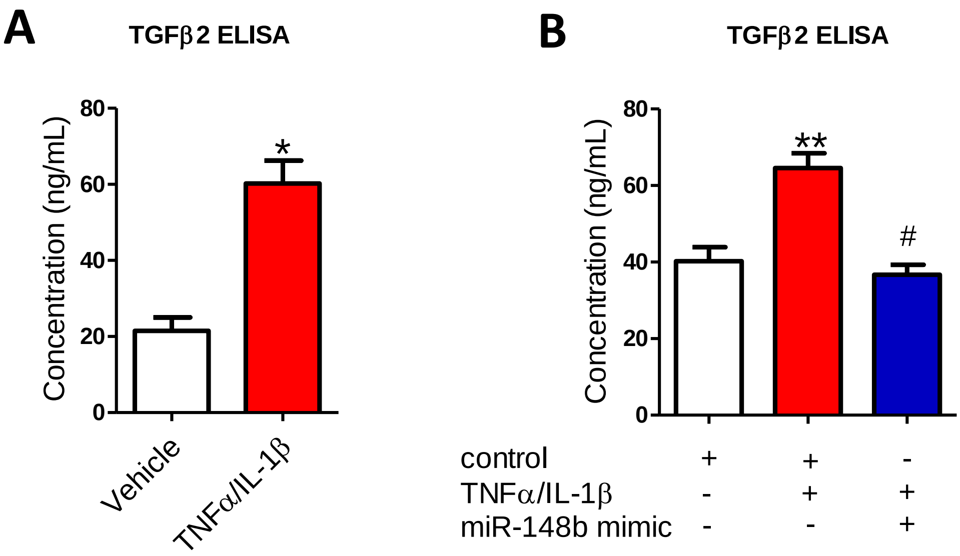

Figure S4. Secreted TGFβ2 following TNFα/IL-1β treatment and miR-148b mimics transfection. **A.** Quantification of TGFβ2 ELISA experiment expressed in TGFβ2 concentration (ng/mL) in TNF-α/IL-1β treated samples vs the control; **B.** Quantification of TGFβ2 ELISA experiment expressed in TGFβ2 concentration (ng/mL) in miR-148b gain-of-function and/or TNFα/IL-1β treated samples vs the control; Values are means±SEM (n=5). *P<0.05; **P<0.01 vs control; . #P<0.05 vs TNFα/IL-1β. Unpaired two-tailed Student's t-test and one-way ANOVA statistical test followed by Bonferroni post-hoc analyses were applied.

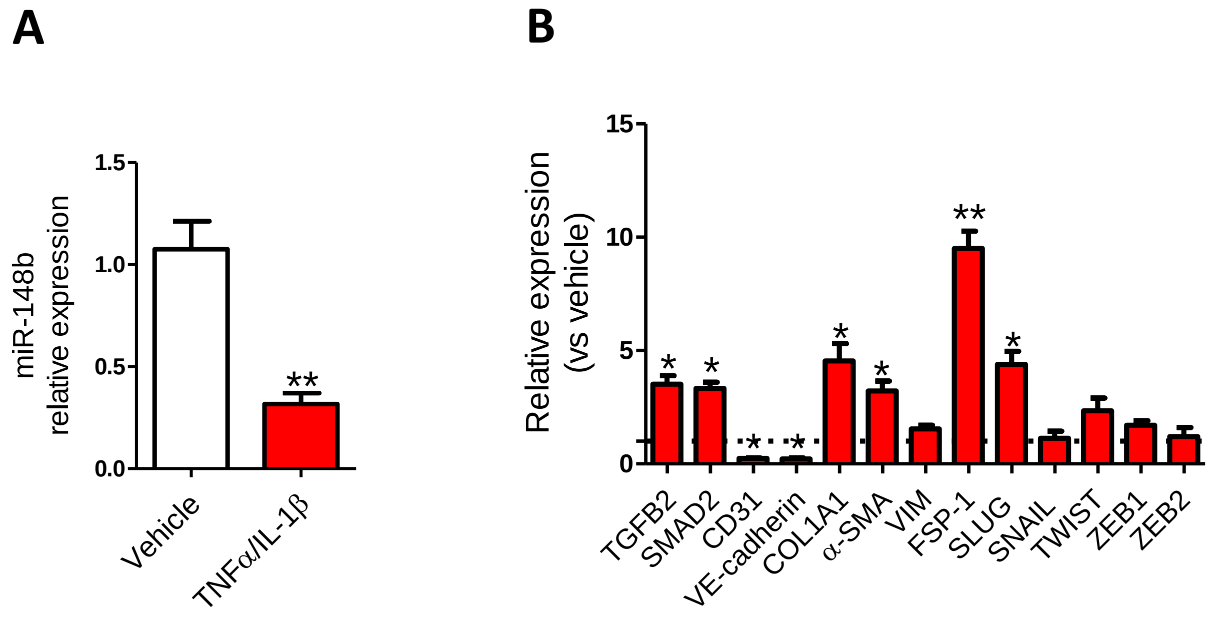

Figure S5: miR-148b EndMT markers expression in ECs after 14 days of cytokine-treatment.
A. Relative miR-148b expression after treatment of HUVECs treated with TNFα/IL-1β for 14 days; **B.** Relative expression of TGFB2, SMAD2, CD31, VE-cadherin, SNAIL, SLUG, TWIST, ZEB1/2, VIM, FSP-1, α-SMA and COL1A1; the dotted line represents relative expression in the vehicle-treated HUVECs (n=3); Values are means±SEM. *$P<0.05$; **$P<0.01$ vs vehicle. Unpaired two-tailed Student's t-test was applied.

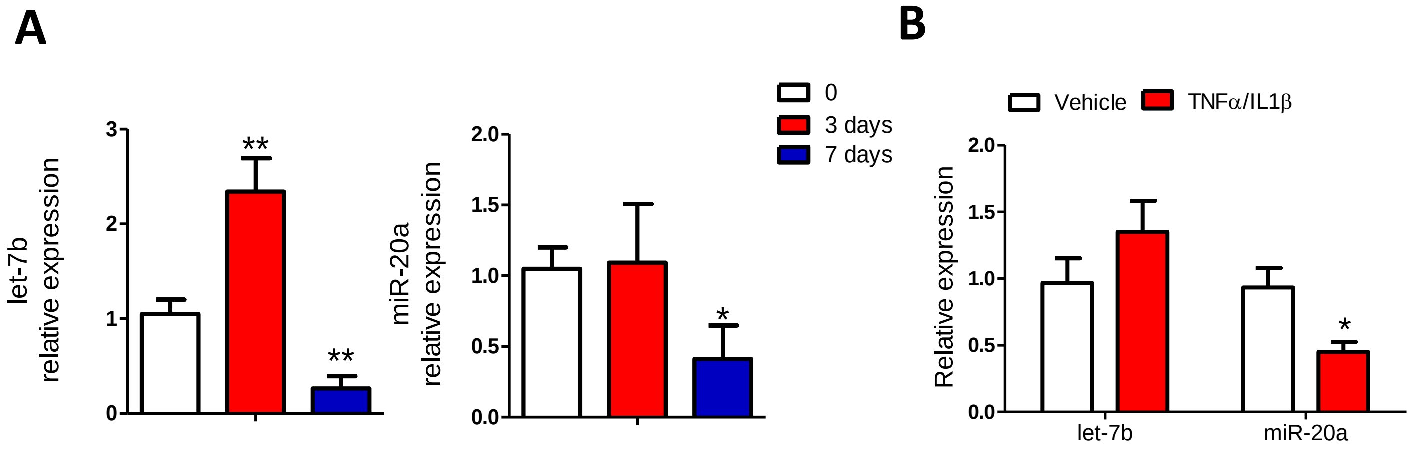

Figure S6: Regulation of let7b and miR-20a expression *in vitro and in vivo*. A. Relative let7-b and miR-20a expression in vivo during wound healing progression and **B.** following TNF-α/IL-1β treatment in ECs Values are means±SEM (n=3). *P<0.05; **P<0.01 vs day 0; . *P<0.05 vs vehicle. Unpaired two-tailed Student's t-test was applied.

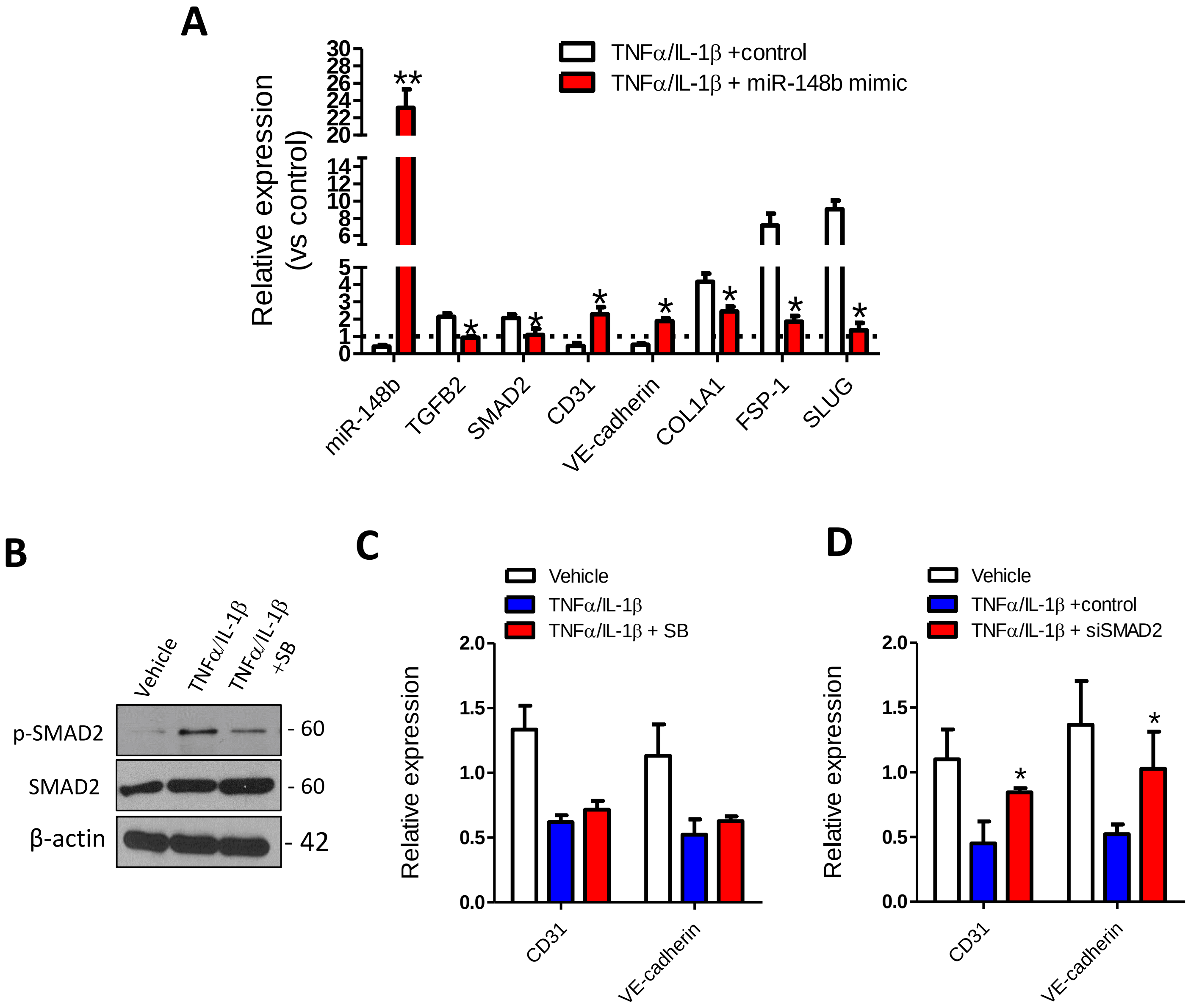

Figure S7: miR-148b mimics and SMAD2 siRNA regulate cytokine-mediated EndMT

HUVECs were transfected with miR-148b mimic, SMAD2 siRNA or control and treated with TNF-α/IL-1β for 6 days. **A.** Relative expression of miR-148b, TGFB2, SMAD2, CD31, VE-cadherin, COL1A1, FSP-1 and SLUG (n=3); **B.** Western blot for phospho-SMAD2, SMAD2 and β-actin in HUVECs treated with TNF-α/IL-1β and ALK5 inhibitor (SB431542; 10μM) for 6 days. **C.** Relative expression CD31 and VE-Cadherin in HUVECs following treatment with ALK5 inhibitor or **D.** SMAD2 siRNA and/or TNFα/IL-1β ; Values are means±SEM (n=3). *P<0.05; **P<0.01 vs TNFα/IL1β + control oligonucleotides. Unpaired two-tailed Student's t-test was applied.

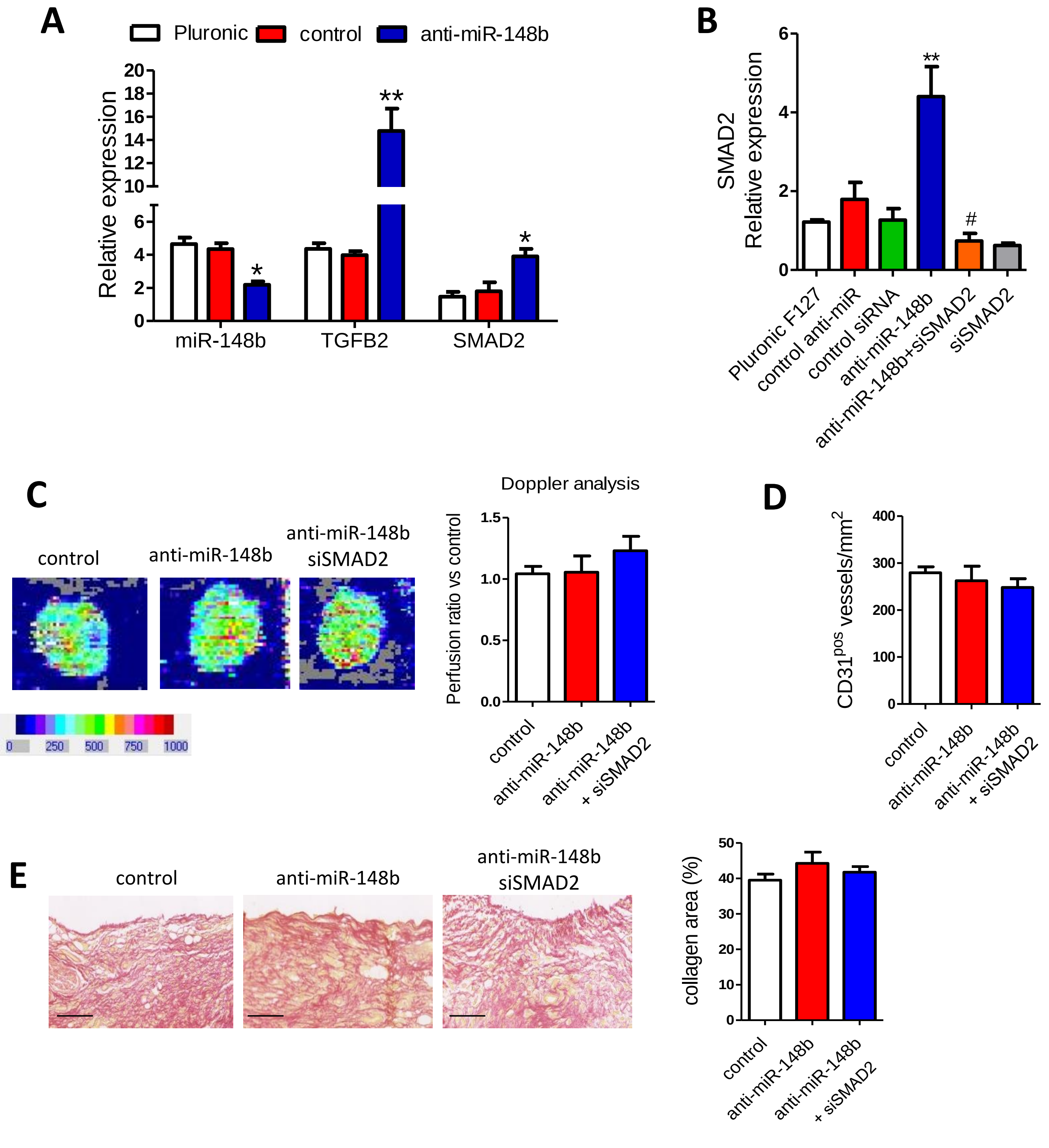

Figure S8: Analysis of anti-miR-148b delivery *in vivo*

Dermal wounds were treated with control and anti-miR-148b or SMAD2 siRNA at 7 days. **A.** Relative expression of miR-148b, TGFB2 and SMAD2 (n=5); **B.** Relative SMAD2 expression in dermal wounds treated (n=5); **C.** *left* Representative colour laser Doppler images are taken at 5 days post wounds. *Right.* Chart shows level of wounds perfusion in mice (calculated as the ratio between treated and control blood flow; n=8 per group). **D**. Quantification of vessel density expressed as CD31 positive vessels/mm2 (n=8); **E.** *left*, Sirius Red staining of wounds obtained at day 7 post wounding; *right*, Collagen quantification. Scale bar represents 100µm (magnification 200x). Values are means±SEM. *P<0.05; **P<0.01 vs control; $^{\#}$P<0.05 vs anti-miR-148b . Unpaired two-tailed Student's t-test and one-way ANOVA statistical test followed by Bonferroni post-hoc analyses were applied.
